# Design, Manufacturing, and Control of a Pneumatic-Driven Passive Robotic Gait Training System for Muscle-Weakness in a Lower Limb

**DOI:** 10.3390/s21206709

**Published:** 2021-10-09

**Authors:** I-Hsum Li, Yi-Shan Lin, Lian-Wang Lee, Wei-Ting Lin

**Affiliations:** 1 Department of Mechanical and Electro-Mechanical Engineering, Tamkang University, New Taipei City 25137, Taiwan; ihsumlee@mail.tku.edu.tw (I.-H.L.); 406370592@o365.tku.edu.tw (W.-T.L.); 2 Department of Mechanical Engineering, National Chung Hsing University, Taichung City 40227, Taiwan; g108061307@mail.nchu.edu.tw

**Keywords:** gait training, rehabilitation robotics, assistive exoskeleton, body weight support system, gait orthosis system, interval type-2 fuzzy sliding control

## Abstract

We designed and manufactured a pneumatic-driven robotic passive gait training system (PRPGTS), providing the functions of body-weight support, postural support, and gait orthosis for patients who suffer from weakened lower limbs. The PRPGTS was designed as a soft-joint gait training rehabilitation system. The soft joints provide passive safety for patients. The PRPGTS features three subsystems: a pneumatic body weight support system, a pneumatic postural support system, and a pneumatic gait orthosis system. The dynamic behavior of these three subsystems are all involved in the PRPGTS, causing an extremely complicated dynamic behavior; therefore, this paper applies five individual interval type-2 fuzzy sliding controllers (IT2FSC) to compensate for the system uncertainties and disturbances in the PRGTS. The IT2FSCs can provide accurate and correct positional trajectories under passive safety protection. The feasibility of weight reduction and gait training with the PRPGTS using the IT2FSCs is demonstrated with a healthy person, and the experimental results show that the PRPGTS is stable and provides a high-trajectory tracking performance.

## 1. Introduction

Walking is an important function for human beings to maintain quality of life. However, many diseases, such as stroke, spinal cord injury, traumatic brain injury, cerebral palsy, and multiple sclerosis, can restrict our independent mobility. Rehabilitation from a walking disability requires much physical therapy. In general, with manual assistance it is very difficult to maintain high-quality therapy with high repeatability and precision across full-gait training sessions and also lacks objective measures of patient performance and progress. A patient needs more than one physiotherapist to perform physical treatments, because the physiotherapists have to support the patient to prevent the knee from buckling during standing, provide additional momentum to maintain the leg’s smooth swing, evaluate correctness of gait movement, and land feet carefully and simultaneously. Studies have shown that continuous passive motion equipment in the first stage of rehabilitation can effectively treat hip and knee joint spasms and contractures [1,2]. An integrated rehabilitation system containing a treadmill, a low-limb exoskeleton, and a body-weight support system can provide continuous passive motion and reduce therapists’ workload [3,4].

Body-weight supported training was first presented in the mid-1980s [5]. Body-weight supported training normally utilizes a harness, cables, pulleys, a frame, and actuators to carry the patient’s body weight. It intends to reduce the demand on the muscles by using a harness to support the patient’s body weight, thus providing more effective and efficient rehabilitation for patients. It also allows individuals to practice walking-like motions repetitively. A passive body-weight support training system, as presented in the 1990s [6,7], uses a series of springs to maintain a constant vertical force on the body and provides manual tuning for force calibration. Some advanced body-weight support training systems can actively provide an unloading force to regulate the patient’s weight [8,9,10,11,12,13]. Active body-weight support training systems exhibit many advantages and have been clinically validated for gait rehabilitation [14,15,16,17,18,19]. Edgerton et al. [13] and Barbeau et al. [17] integrated body-weight support training with a postural supporter to provide postural support, thus increasing patients’ confidence during ambulation practice. Gazzani et al. [20] presented a pneumatic body-weight support training system. They used a pneumatic cylinder to drive a cart rolled along a track mounted on a frame. The pneumatic actuator provides constant feedback unloading force to compensate for output error and keeps the system stable. Nevertheless, this body-weight support training system cannot dynamically regulate the output force during gait training.

Robot gait rehabilitation systems present a new rehabilitation method for patients. They provide overall controllable-level assistance for gait rehabilitation, allowing repetitive and task-specific training, and their sensor data help therapists to quantify impairment severity and recovery for patients. They also have great potential to reduce therapist workload and therapy costs. McDaid et al. [21] presented a robotic exoskeleton for improving lower-limb gait rehabilitation. This device is light and compact and can completely fit onto patients’ bodies. Lokomat [22] is a well-known robot rehabilitation device. It uses a BWST to decrease patient’s weight and applies a robotic exoskeleton to assist basic walking functions. However, Lokomat is very expensive to purchase and maintain. LOPES [23] uses a servo motor to drive a flexible Bowden transmission cable and a series of elastic elements to suspend a patient. It allows stiff control along a given joint trajectory of an entire gait training cycle. Compared with Lokomat, LOPES is more flexible and comfortable in use. The Auto Ambulator [24], developed by the US Encompass Health Corporation, provides a harness and an overhead hoist to suspend a patient above a treadmill and uses robotic arms to strap the patient’s legs and drive them. In gait rehabilitation training, Guo et al. [25] proposed human–robot interactive control for a lower limb exoskeleton robot. Each lower limb exoskeleton has two rotational degrees of freedom and is driven by pneumatic, proportional valves. Considering its suitability, they used an adaptive admittance model to adapt it for human–robot interactions. They demonstrated that the flexibility of the pneumatic actuators and compliance provided by the controller could contribute to the training comfort, safety, and therapeutic outcome in gait rehabilitation. Beyl et al. [26] used a pleated pneumatic muscle to develop a single-joint powered knee exoskeleton, named KNEXO. They presented a soft controller, called a Proxy-Based Sliding Mode Control (PSMC), for a pneumatic-driven joint to increase safety for users. The PSMC can provide KNEXO with a smooth force to overdamped recovery from large tracking errors caused by abnormal events and can ensure tracking performance. However, it is lacking in theoretical demonstration and has difficulties in adapting to environmental changes, i.e., the coupling dynamics. Among robot gait rehabilitation systems, the pneumatic-driven robot gait rehabilitation system [25,26,27,28,29,30] can provide many advantages over motor-driven ones: (1) the manufacturing cost is lower because the pneumatic-driven robot gait rehabilitation system is driven by low-cost pneumatic actuators, (2) the pneumatic-driven robot gait rehabilitation system is easy to maintain and keep clean, and (3) the pneumatic-driven robot gait rehabilitation system provides compliance movement and reactions. However, the pneumatic-driven robot gait rehabilitation system exhibits very complicated motion and is difficult to mathematically model, because its dynamics are related to the air pressure, load changes, temperature changes, and external disturbances. In practice, using a proportional-integral-derivative controller (PID-controller) is a common approach in the industry. However, it has poor robustness against disturbances and system uncertainties, and has to compromise between rapid response and small tracking error. Instead of using a PID controller, intelligent control and nonlinear control have shown significant improvements in pneumatic-driven systems’ robustness and stability. Among them, an interval type-2 fuzzy controller [31,32,33,34] was presented to increase system robustness and reliability when subject to system modeling uncertainties, measurement noise, and external disturbances. Chiu et al. [35] used an interval type-2 fuzzy controller to maintain the stability of a single-wheel vehicle while a person was standing on it. Kelekci et al. [36] presented a real-time interval type-2 fuzzy controller for trajectory and vibration control of a flexible joint manipulator and proved overall system stability using the Lyapunov stability theorem. In 2016, we presented an interval type-2 fuzzy controller with an adaptive fuzzy sliding compensator [37] for a pneumatic-drive active suspension system to compensate road disturbances and improve ride comfort.

This study aims to develop a pneumatic-driven robotic passive gait training system (PRPGTS) for individuals who need gait rehabilitation but suffer from weakened lower limbs. The PRPGTS is designed as a pneumatic-driven system with three subsystems: a pneumatic body-weight support system (PBWSS), a pneumatic postural support system, and a pneumatic gait orthosis system (PGOS). The motion of the PRPGTS contains three parts, and they all influence each other, implying that the dynamics of the PRPGTS is coupled and complicated. This leads to the problem of designing a controller for the PRPGTS. In this study, we separately design a compensator for each subsystem, and the influences of one on another are viewed as uncertainties in controller design. As the PBWSS must be able to accommodate people of various weights, this study uses an IT2FSC to achieve the desired reduction ratios for patients. The PGOS is subject to an external load due to the human–robot interaction during the gait training, so this study uses four IT2FSCs with the pulse-width modulation control to compensate for unmodeled dynamics. The contributions in this paper include:

1. The PRPGTS is a self-designed and-manufactured gait training system. It features a PBWSS, a pneumatic postural support system, and a PGOS. The PBWSS and the pneumatic postural support system, respectively, provide weight support and postural support for a patient during gait training, and the PGOS drives the patient’s legs to follow a pre-set training gait cycle;
2. The PRPGTS is a pneumatic-driven system. It can significantly reduce manufacturing costs and provide compliance force;
3. The PRPGTS has approval for experimental testing on healthy individuals from the Fu Jen Catholic University Institutional Review Board (IRB);
4. The IT2SFCs are designed for the PRPGTS to provide excellent robustness against uncertainties and external loads. In this paper, four interval type-2 fuzzy sliding pulse-width modulation controllers are designed to regulate the four joints of the PGOS and achieve precise and stable trajectory tracking control, and one IT2SFC is used to regulate the supporting force for the PBWSS;
5. The experimental results demonstrate that the PRPGTS provides a stable control force to assist a subject in gait training and gives assist-as-needed PBWSS and pneumatic postural support system during gait training.

The remainder of this paper is organized as follows. Section 2 describes the designed pneumatic robot gait training system’s design, hardware configuration, and functions. Section 3 details the method for extracting the gait-training trajectory. Section 4 describes the controller design for the pneumatic body-weight support system and pneumatic gait orthosis system. Section 5 presents the results of experiments conducted to verify the feasibility of the designed pneumatic robot gait training system. Finally, Section 6 presents the conclusions of this study.

## 2. Prototype of the Pneumatic-Driven Robotic Passive Gait Training System

**Figure 1:**
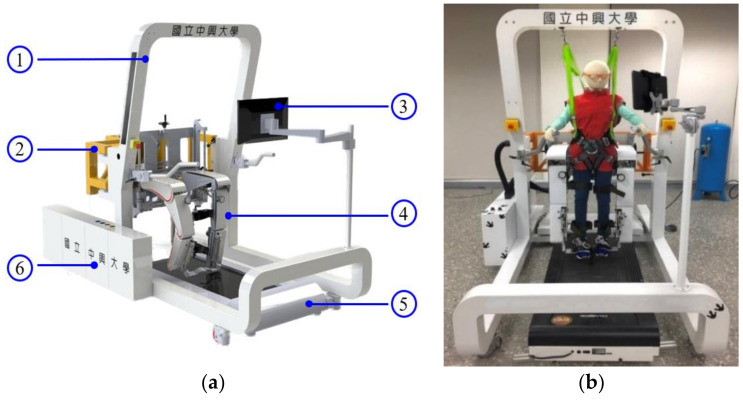
Prototype of the PRPGTS. (**a**) illustrates the prototype of the PRPGTS; (**b**) photo of the PRPGTS. ➀ PBWSS, ➁ pneumatic postural support system, ➂ human–machine interface, ➃ PGO, ➄ treadmill, ➅ embedded controller.

A pneumatic actuator has a simple mechanical structure: a cylinder with a piston, allowing compressed air to push a piston and produce mechanical motion. It also has excellent damper–spring characteristics and can provide compliance and continuous force to the PRPGTS. Hence, the pneumatic-driven PRPGTS can provide patients with a more comfortable training experience than other drivers, i.e., motors. Figure 1a illustrates the prototype of the PRPGTS and Figure 1b shows a photo. The PRPGTS comprises three pneumatic subsystems:

- the pneumatic body-weight support system (PBWSS),
- the pneumatic postural support system, and
- the pneumatic gait orthosis system (PGOS).

In addition, the PRPGTS uses an FPGA embedded controller to operate these three subsystems and has a human–machine interface to provide machine–human interaction. This section presents the design and the electro-mechanics of the PBWSS, the pneumatic postural support system, and the PGOS in detail.

### 2.1. Pneumatic Body-Weight Support System

**Figure 2:**
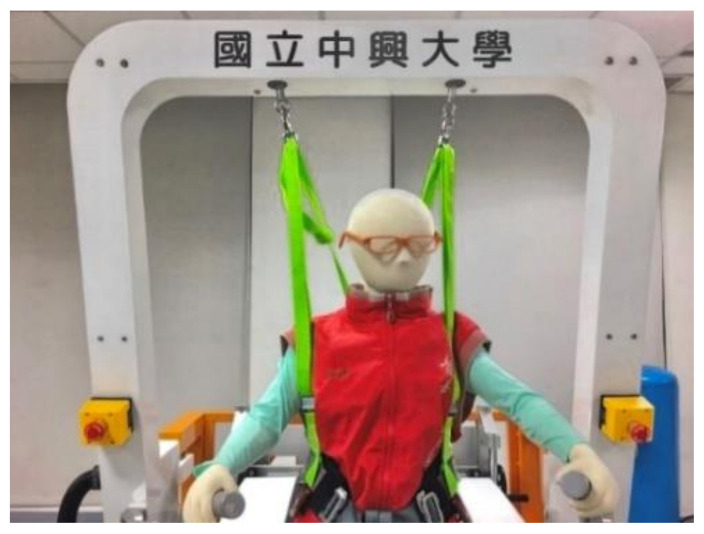
Photo of the PBWSS.

**Figure 3:**
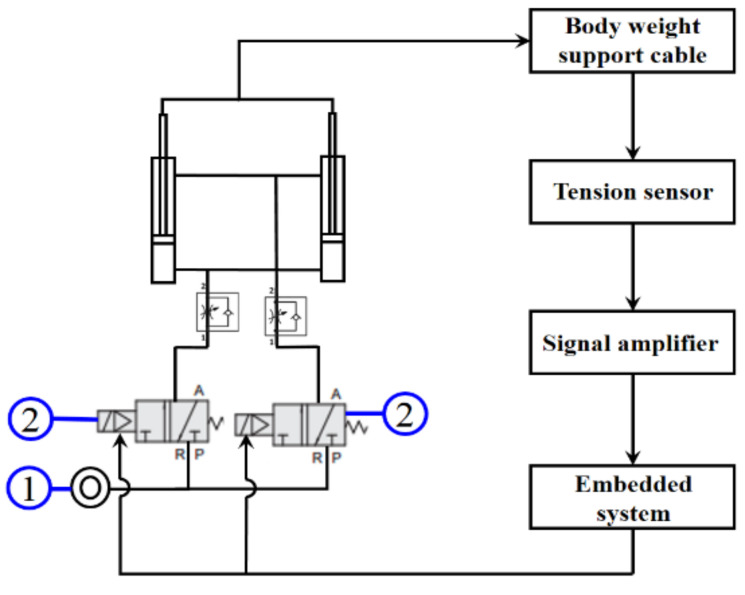
Overall Control System of the PBWSS. ➀ Air source, ➁ Proportional pressure control valve.

Figure 2 shows a photo of the PBWSS and Figure 3 illustrates its overall control block. The PBWSS uses two pneumatic actuators (CHELIC SDA40–300); one with a 40-mm diameter and the other with a 300-mm stroke. The PBWSS is set to provide different weight reductions: 20%, 30%, and 40%. Here, we chose force feedback control to regulate the PBWSS, because it can directly and precisely supply force change in the same ratio as a change in the input voltage. A proportional pressure control valve (FESTO VPPM-6L-L-1-G18–0L6H-V1P-S10) is applied to regulate compressed air for the PBWSS; it can regulate the pressure inside a pneumatic actuator within the interval of [0.06bar 6bar], which is directly proportional to the voltage between 0 v to 10 v. The PBWSS suspends a patient using two harnesses. The harnesses are connected to two pneumatic actuators through two load cells (Memstec S-100); thus, the force exerted on the patient can be measured. The force measurement is amplified by an amplifier of Memstec JS-101 and is sent to the embedded controller.

### 2.2. Pneumatic Postural Support System

**Figure 4:**
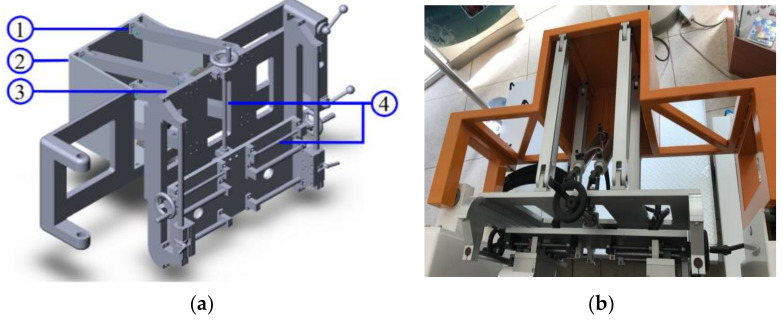
(**a**) The design of the pneumatic postural support system; (**b**) picture of the pneumatic postural support system. ➀ Hinge, ➁ Back frame of revolving door, ➂ Pneumatic cylinder, ➃ Lead screw.

The pneumatic postural support system is designed to balance the patient’s body during gait training. The pneumatic postural support system is a passive pneumatic system and has a natural spring-damper characteristic to buffer external forces when the patient’s center-of-gravity changes during gait training. Figure 4 shows a CAD picture and a photo of the pneumatic postural support system. The pneumatic postural support system contains two parts: a quadrilateral frame applied to support the patient’s pelvis, and two pneumatic actuators applied to produce force. The quadrilateral frame has a revolving door and a back frame. The revolving door is connected to the back frame with hinges, so the quadrilateral frame is adjustable and rotatable to allow patients to use it easily. Here, the two pneumatic actuators of the pneumatic postural support system have a 40-mm diameter and 250-mm long stroke.

### 2.3. Pneumatic Gait Orthosis System

**Figure 5:**
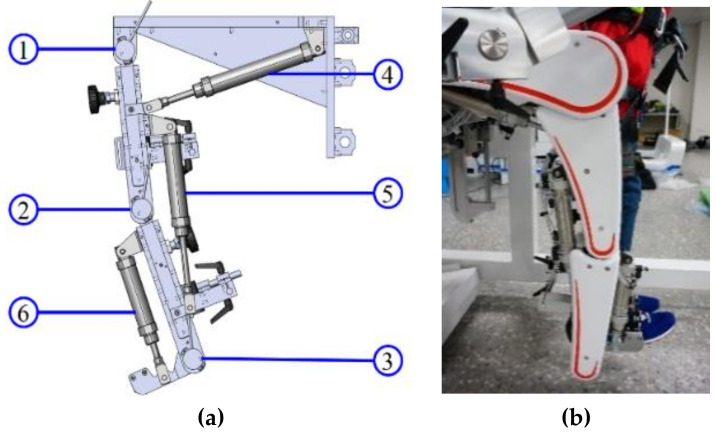
(**a**) The design of the PGOS; (**b**) picture of the PGOS. ➀ Hip joint encoder, ➁ Knee joint encoder, ➂ Ankle joint encoder, ➃ Hip joint pneumatic cylinder,➄ Knee joint pneumatic cylinder, ➅ Ankle joint pneumatic cylinder.

It is very challenging to design a lower limb exoskeleton that allows functional gait training, because the design of the PGOS must be flexible enough to provide functional motion and allow a patient to walk normally and safely when the patient wears the exoskeleton. Moreover, the PGOS has to be light, easy to wear, and safe and comfortable in use. Designed to have these characteristics, the PGOS uses six pneumatic actuators to form a three-rotation DOFs structure for each leg, and the frame of the leg is constructed using three pieces of aluminum, which are light and have excellent strength. The length of the lower limb exoskeleton can be adequately adjusted to fit the shape of patients. Figure 5a shows a CAD picture of the PGOS, while Figure 5b shows a photo of the PGOS. Table 1 shows the specifications of the pneumatic cylinders and the valves for the PGOS. Three encoders (ELCO E38F8-C4AR-2000) are attached to the hip, knee, and ankle joints for each leg, as shown in indicators 1, 2, and 3 of Figure 5a. The resolution of the encoders is 0.045 degrees. Here, we chose an on-off valve for the PGOS to regulate compressed air because it is much cheaper than a servo valve. In general, the price of an on-off valve is one-twentieth of a servo valve. In practice, an on-off valve can achieve accurate positional control if given a proper pulse-width modulation signal.

## 3. Design of the Gait Training Trajectory for the PGOS

This section shows the forward kinematic model of the PGOS and clearly gives the steps of extracting gait training trajectories from a healthy person. Section 3.1 shows the D-H table of the PGOS; Section 3.2 shows the procedures for capturing joint motions from a healthy person using a Kinematracer [38]. Section 3.3 describes how we determine a full training gait cycle. The left- and right-limb mechanisms of the PGOS are identical, apart from a phase difference of 180°. For simplicity, this study uses the right limb to demonstrate the forward kinematic motion.

### 3.1. Forward Kinematic Analysis of the PGOS

**Figure 6:**
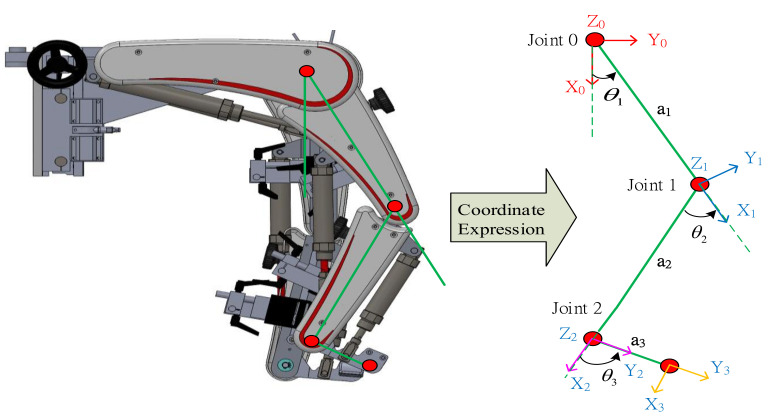
Coordinate system of the lower exoskeleton.

The PGOS has six joint variables for two limb exoskeletons. One of them is designed as having three rotational degrees of freedom, in which the hip and knee joint variables are controllable, and the ankle joint variable is set to be a fixed angle during gait training. All of the rotation axes are located on the joints and are perpendicular to the others. The coordinate system of the lower-limb exoskeleton is illustrated in Figure 6, where three coordinates, ($X_{0},Y_{0},Z_{0}$), ($X_{1},Y_{1},Z_{1}$), and ($X_{2},Y_{2},Z_{2}$), are respectively represented as the locations of the end-effecters for the three links. The world reference coordinate was chosen as the first joint with the coordinate ($X_{0},Y_{0},Z_{0}$), in which the $X_{0}$ axis runs in a positive direction to the ground, the $Y_{0}$ axis runs in a positive direction to the front, and the $Z_{0}$ axis runs in a positive direction to the side. The Denavit–Hartenberg coordinates corresponding to each rotation joint are shown in Table 2. In Figure 6, $a_{i}$ is the distance between $Z_{i-1}$ and $Z_{i}$ in the direction of $X_{i}$, $\alpha_{i}$ is the angle between $Z_{i-1}$ and $Z_{i}$ in the direction of $X_{i}$, $d_{i}$ is the distance between $X_{i-1}$ and $X_{i}$ in the direction of $Z_{i}$ and $\theta_{i}$ is the angle between $X_{i-1}$ and $X_{i}$ in the direction of $Z_{i}$. $d_{i}$ is zero and $\alpha_{i}$ is zero of the PGOS.

According to the Denavit–Hartenberg Table, the Denavit–Hartenberg matrix from the *i*th joint to *(i-1*)th joint is:

(1) Tii−1=cosθi−sinθicosαisinθisinαiaicosθisinθicosθicosαi−cosθisinαiaisinθi0sinαicosαidi0001

and the Denavit–Hartenberg matrix T30=T10T21T32 is calculated by:

(2) T30=T10T21T32=cosθ1−sinθ10a1cosθ1sinθ1cosθ10a1sinθ100100001cosθ2−sinθ20a2cosθ2sinθ2cosθ20a2sinθ200100001cosθ3−sinθ30a3cosθ3sinθ3cosθ30a3sinθ300100001=c123−s1230a3c123+a2c12+a1c1s123c1230a3s123+a2s12+a1s100100001

where $c_{i}=\cos\theta_{i}(i=1,2,3)$, $s_{i}=\sin\theta_{i}(i=1,2,3)$, $c_{12}=\cos(\theta_{1}+\theta_{2})$, $c_{123}=\cos(\theta_{1}+\theta_{2}+\theta_{3})$, $s_{12}=\sin(\theta_{1}+\theta_{2})$, and $s_{123}=\sin(\theta_{1}+\theta_{2}+\theta_{3})$. According to the definition of the transformation matrix, the fourth column of Equation (2) yields the position of the end-effector $\left(p_{x},p_{y},p_{z}\right)$ of the PGOS with respect to the reference frame $\left(X_{0},Y_{0}{,Z}_{0}\right)$, as:

(3) $$p_{x}=a_{3}c_{123}+a_{2}c_{12}+a_{1}c_{1},\;p_{y}=a_{3}s_{123}+a_{2}s_{12}+a_{1}s_{1},\;p_{z}=0.\;$$

**Figure 7:**
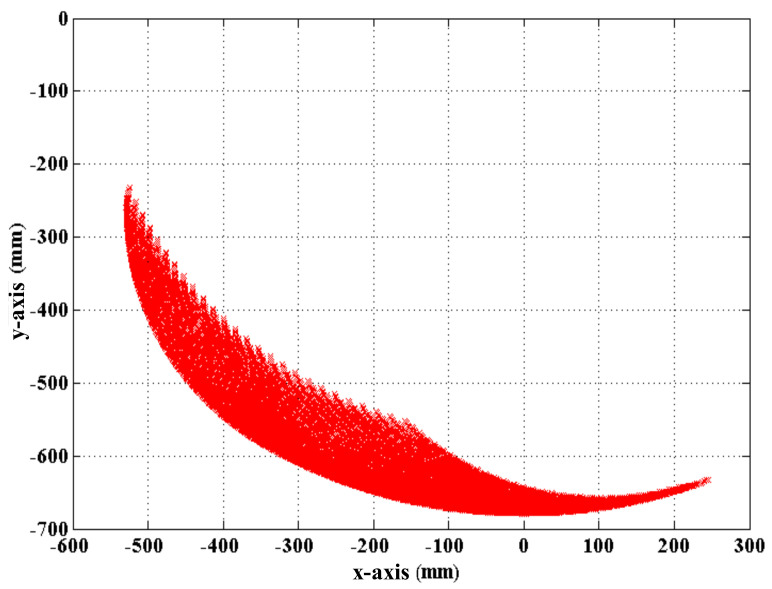
Motion space simulation of the lower limb exoskeleton.

In Equation (3),$p_{z}$ is zero because the lower-limb exoskeleton cannot move laterally. Figure 7 shows that the PGOS covers the area from −530 mm to 250 mm in the *y*-axis direction and from −220 mm to −690 mm in the *x*-axis direction.

### 3.2. Gait Parameter Extraction

**Figure 8:**
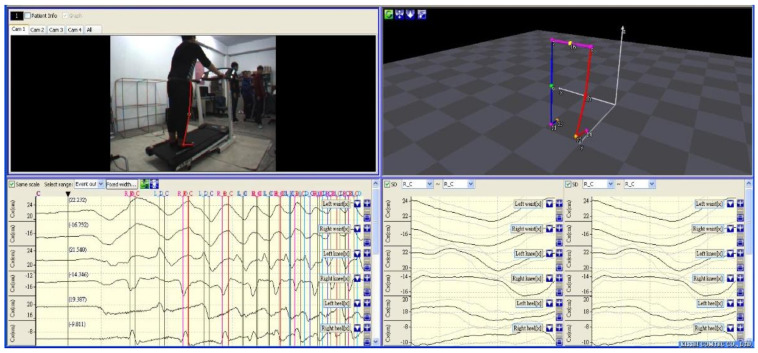
Process of capturing a gait cycle when a person walks in a 3D space.

Determining a proper training cycle is crucial for PGOS gait training. In general, a full gait cycle is defined as a progression of motion whereby one leg returns to a specific position during walking. Hence, to provide an appropriate reference gait, this study uses a Kinematracer [39,40] to capture a full gait training cycle from a healthy male walking on a treadmill at a constant speed of 1.5 km/h. As illustrated in Figure 8, eight lighting balls were attached to the healthy male’s left and right hip, knee, and ankle joints during the process; while the male walked on the treadmill, the Kinematracer used four high-speed cameras around the treadmill to capture images. The eight lighting balls’ spatial coordinates and angle variations were identified by image processing from the image, which reflects the male’s gait cycle; after that, all of the information (the coordinate and the angle of the left and right hip, knee, and ankle joints) was digitalized and stored in a computer. The right figure of Figure 8 shows the process of capturing the gait cycle when a male walks in a 3D space.

### 3.3. Design of Gait Training Cycle

**Figure 9:**
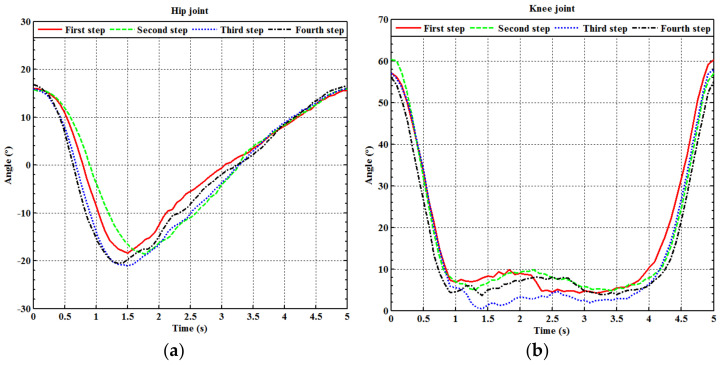
Fitting curves of full gait training cycle. (**a**) The hip of the right leg; (**b**) the knee of the right leg.

A curve fitting method [41] is used to build a continuous full gait training cycle from the captured digital information for the hip and knee’s joints. It can be described as:

(4) $$f(t)=k_{1}\times e^{b_{1}\times \cos(t+c_{1})}+k_{2}\times e^{b_{2}\times \cos(t+c_{2})}+k_{3}\times e^{b_{3}\times \cos(t+c_{3})}+k_{4},$$

where $k_{1},\;k_{2},\;k_{3}$ and $k_{4}$ are real numbers. As the two legs describe a very similar motion, we adopted the right leg movement as the reference gait cycle. Four full gait cycles, as described by Equation (4), in five seconds are shown in Figure 9a (for the hip) and Figure 9b (for the knee) for the right leg. Note that the angle of the ankle joint is fixed at 90 degrees during the gait training. An average of the four gait cycles gives two continuous rotation angle functions for the hip and knee, respectively, which are

(5) $$f_{hip}(t)=-12.523e^{0.983\cos(t+4.714)}+32.324e^{-0.297\cos(t+2.865)}+0.343e^{3.201\cos(t-0.521)}-19.856,\;\mathrm{for}\;\mathrm{the}\;\mathrm{hip},$$

and

(6) $$f_{knee}(t)=-0.665e^{3.223\cos(t+0.485)}+0.241e^{-2.561\cos(t-0.500)}+1.860e^{3.579\cos(t+0.123)}+3.866,\;\mathrm{for}\;\mathrm{the}\;\mathrm{knee}.$$

**Figure 10:**
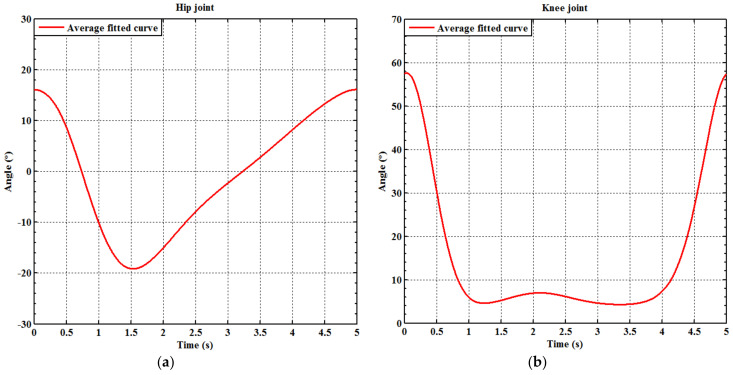
Average of the fitting curves of the full gait training cycle. (**a**) The hip of the right leg; (**b**) the knee of the right leg.

**Figure 11:**
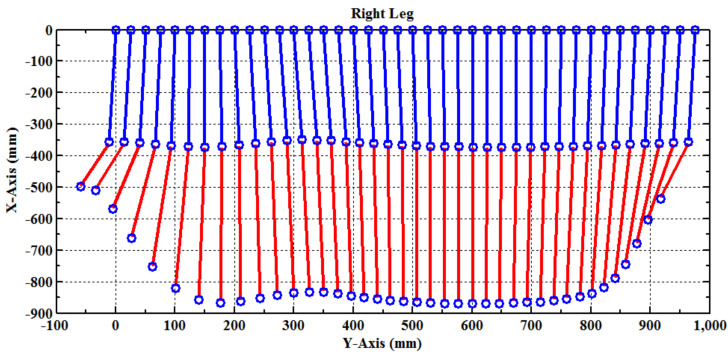
Examination of the full gait training cycle for the right leg.

Figure 10a,b, respectively, show the fitting curves for the hip and knee in five seconds, which was set as a standard reference gait training cycle for the experiments. The duration of the gait training cycle is adjustable. For example, one can double the duration of the standard reference gait training cycle to ten seconds. The PGOS is a pneumatic driven system with four translational motions, so Equations (5) and (6) have to be transferred to translational motion. Clearly, the rotational motion and the translational motion have linear relationships, which are

(7) $$y_{hip}(t)=\kappa f_{hip}(t),\;\mathrm{for}\;\mathrm{the}\;\mathrm{hip},$$

and

(8) $$y_{knee}(t)=\kappa f_{knee}(t),\;\mathrm{for}\;\mathrm{the}\;\mathrm{knee},$$

where κ is a positive constant. Figure 11 shows the examination for the full gait training cycle calculated by the forward kinematic Equation (3), giving the angle variations of the hip and knee (described by (5) and (6)). The blue line represents the link from the hip joint to the knee joint, and the red line represents the link from the knee joint to the ankle joint.

## 4. Controller Design for the PRPGTS

The PRPGTS is driven by ten pneumatic actuators to drive a gait training cycle. It uses two pneumatic actuators to provide force for the pneumatic postural support system; two for the PBWSS, and six for the PGOS. The PPSS is built to be a passive pneumatic-driven system, and the PBWSS and the PGOS are designed as active pneumatic-driven systems. The PGOS uses four pneumatic actuators for the hip and the knee joints to create gait-training motions and uses two pneumatic actuators to provide a constant force and maintain a consistent angle for the ankle joint. A pressure control proportional valve is applied for the PBWSS to regulate force and provide body-weight-support for the patient, and four fast switching on-off valves are used for the PGOS to drive a full gait training cycle. Since these two kinds of valves yield different output signals and are excited by different input signals, this study presents two types of the IT2FSC [36], to compensate for the uncertainties and provide stable gait training for the PBWSS and the PGOS, respectively.

### 4.1. Mathematical Model of the Pneumatic Actuator

**Figure 12:**
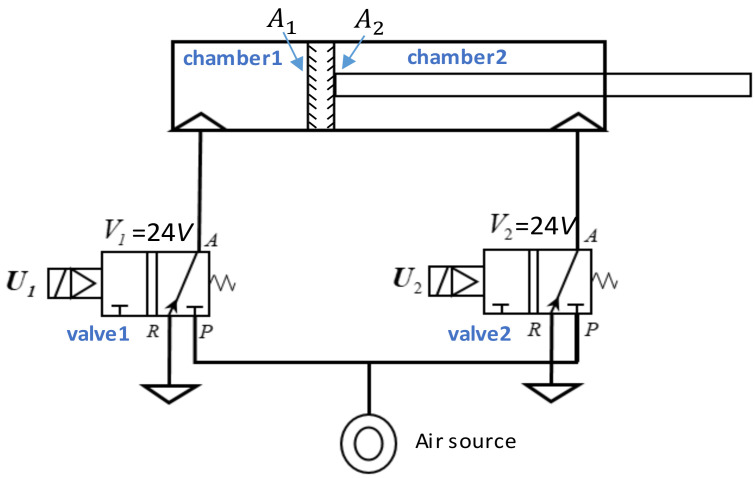
Diagram of a double-acting pneumatic cylinder control.

Figure 12 shows a diagram of a double-acting pneumatic cylinder with two 3/2 way pneumatic solenoid valves, where point A is an inlet of air, point R is an exhaust of air, point P is an air source, $U_{i}(i=1,2)$ are control signals, and $V_{i}(i=1,2)$ are input voltages, $A_{1}$ and $A_{2}$, respectively, represent the area of the left and right surface of the piston. If valve 1 turns on and valve 2 turns off, air acts on the $A_{1}$ and pushes the piston to the right; with the contrary, the piston moves to the left.

Equation (9) expresses the motion of a pneumatic cylinder as a second-order differential equation:

(9) $$A_{1}\left(P_{1}-P_{2}\right)-A_{2}\left(P_{1}-P_{2}\right)\;=m\frac{d^{2}y}{dt^{2}}+f\frac{dy}{dt}+F_{f}+F_{L},$$

where $P_{1}$ (unit: *n*) is the pressure inside the chamber 1, $P_{2}$ (unit: *n*) is the pressure inside the chamber 2, m (unit: kg) is the lumped mass of the piston, f is the viscous damping coefficient, $F_{f}$ (unit: *n*) is the friction inside the cylinder, $F_{L}$ (unit: *n*) is the sum of the external force, and y is the moving distance of the piston.

### 4.2. Interval Type-2 Fuzzy Sliding Pulse-Width Modulation Control for the PGOS

**Figure 13:**
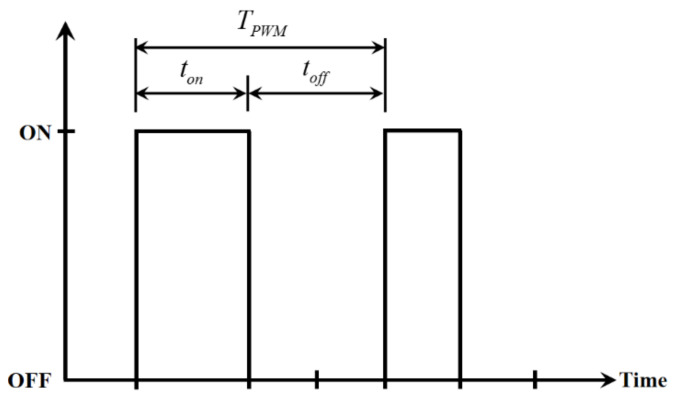
Schematic diagram of the interval type-2 fuzzy sliding pulse-width modulation control.

The proportional directional control valve has a simple dynamic behavior and can provide airflow control at high precision. However, it is costly. A fast switching on-off value is cheap and has a simple mechanical structure, but its dynamics are intrinsically nonlinear. Fortunately, a fast switching on-off valve presents an almost linear dynamic behavior if it is excited by a pulse-width modulation signal. Under comprehensive consideration, the PGOS uses a fast switching valve to regulate the compressed air that flows into a cylinder and uses interval type-2 fuzzy sliding pulse-width modulation control to overcome system nonlinearity and uncertainty. Figure 13 shows a series of on/off pulse-width modulation signals, where $T_{PWM}$ is a period of the carrier wave, $t_{on}$ is the time when the power turns on, called a duty cycle, and $t_{off}$ is the time when the power turns off.

**Figure 14:**
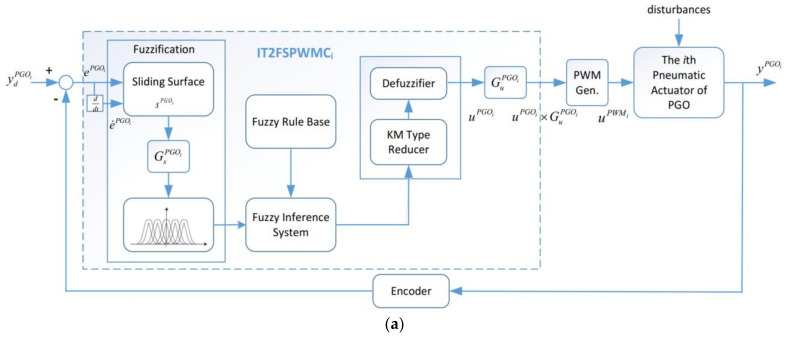

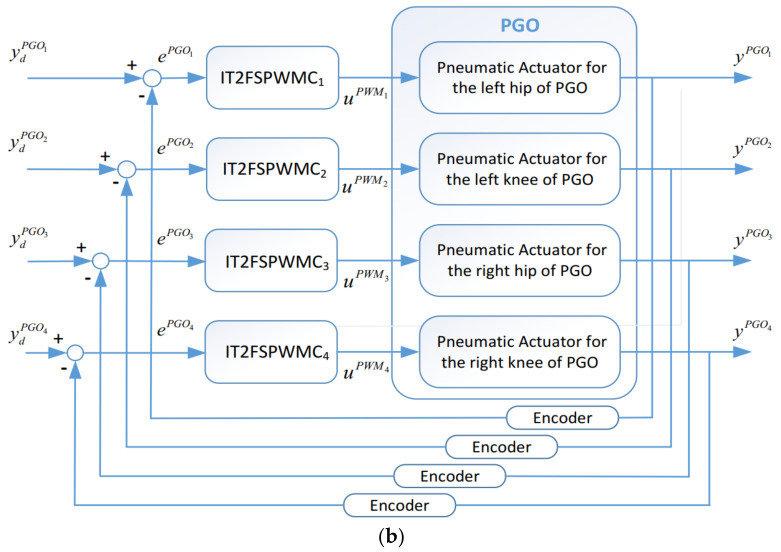
Block diagram of the interval type-2 fuzzy sliding pulse-width modulation control for the PGOS. (**a**) The *i*th interval type-2 fuzzy sliding pulse-width modulation controller; (**b**) the overall control block for the PGOS.

A PGOS will probably encounter external disturbances and system uncertainties during gait training. It is noted that the external disturbances mainly include a patient’s bodyweight and the effects from other pneumatic actuators and the system uncertainties mainly include unmodeled system dynamics. To attenuate these disturbances and uncertainties, the interval type-2 fuzzy sliding pulse-width modulation control is designed as an intelligent and robust compensator, as shown in Figure 14. Figure 14a shows the diagram block of the interval type-2 fuzzy sliding pulse-width modulation control for the *it*h pneumatic cylinder of the PGOS. It can process a sliding surface and fuzzy inference system and output a pulse-width modulation duty cycle. For the *i*th pneumatic actuator control, $y^{PGO_{i}}$ and ${\dot{y}}^{PGO_{i}}$ are the system output and its derivative over time, and $y_{d}^{PGO_{i}}$ and ${\dot{y}}_{d}^{PGO_{i}}$, respectively, stand for the reference input and its derivative over time. The sliding surface of the interval type-2 fuzzy sliding pulse-width modulation control for the *i*th pneumatic cylinder of the PGOS is defined as:

(10) $$S^{PGO_{i}}(t)=\lambda_{1}^{i}e^{PGO_{i}}(t)+\lambda_{2}^{i}{\dot{e}}^{PGO_{i}}(t),$$

where $e^{PGO_{i}}(t)=y^{PGO_{i}}(t)-y_{d}^{PGO_{i}}(t)$, ${\dot{e}}^{PGO_{i}}(t)={\dot{y}}^{PGO_{i}}(t)-{\dot{y}}_{d}^{PGO_{i}}(t)$, and $c_{j}^{i}$ are specified such that $\sum_{j=1}^{2}c_{j}^{i}\lambda_{i}^{j-1}$ is a Hurwitz polynomial. $\lambda_{i}^{j-1}$ can be a different value between the four IT2FSPWMCs. The lth fuzzy rule for the *i*th interval type-2 fuzzy sliding pulse-width modulation control of the PGOS is:

(11) $$R^{l}:if\;S^{PGO_{i}}\;\mathrm{is}\;f_{s}^{l}\;then\;u^{PGO_{i}}\;\mathrm{is}\;f_{u}^{l}\;,\;(l=1,\ldots ,M),$$

where $f_{s}^{l}$ is an interval type-2 fuzzy set and $f_{u}^{l}$ is an interval type-2 singleton fuzzy set. Please note that $f_{s}^{l}$ and $f_{u}^{l}$ can be different fuzzy sets between the four interval type-2 fuzzy sliding pulse-width modulation controllers. The output of the interval type-2 fuzzy sliding pulse-width modulation control is calculated using singleton fuzzification; the product inference and the center-average defuzzification is given as:

(12) $$u^{PGO_{i}}(S^{PGO_{i}},\alpha )=\frac{y_{l}^{i}+y_{r}^{i}}{2}=\frac{1}{2}\left[\alpha_{l}^{T}\alpha_{r}^{T}\right]\left[\begin{array}{l} \xi_{l}\\ \xi_{r} \end{array}\right]=\alpha^{T}\xi ,$$

where $u^{PGO_{i}}$ is the output of the *i*th interval type-2 fuzzy sliding pulse-width modulation control, $y_{l}^{i}$ and $y_{r}^{i}$, respectively, represent the farthest left and the farthest right points of the interval type-2 fuzzy set for the *i*th interval type-2 fuzzy sliding pulse-width modulation control. $\alpha^{T}=\left[\alpha_{1},\alpha_{2},\cdots ,\alpha_{2M}\right]$ is a weight vector. The KM algorithm [40] is used for the type reducer. The farthest left point for the interval type-2 fuzzy set is defined as:

(13) $$y_{l}^{i}=\frac{\sum_{k=1}^{L}{\bar{\mu }}_{f_{S}^{k}}(S^{PGO_{i}})\alpha_{l}^{k}+\sum_{k=L+1}^{M}{\underline{\mu }}_{f_{S}^{k}}(S^{PGO_{i}})\alpha_{l}^{k}}{\sum_{k=1}^{L}{\bar{\mu }}_{f_{S}^{k}}(S^{PGO_{i}})+\sum_{k=L+1}^{M}{\underline{\mu }}_{f_{S}^{k}}(S^{PGO_{i}})}=\sum_{k=1}^{L}{\bar{p}}_{l}^{k}\alpha_{l}^{k}+\sum_{k=L+1}^{M}{\underline{p}}_{l}^{k}\alpha_{l}^{k}=[\begin{array}{cc} {\bar{\alpha }}_{l}^{T} & {\underline{\alpha }}_{l}^{T} \end{array}]\left[\begin{array}{c} {\bar{p}}_{l}\\ {\underline{p}}_{l} \end{array}\right]=\alpha_{l}^{T}\xi_{l},$$

where ${\bar{\mu }}_{f_{S}^{k}}$ and ${\underline{\mu }}_{f_{S}^{k}}$, respectively, represent the upper and lower degrees of the membership function, $\alpha_{l}^{k}$ is the farthest left point of ${\bar{p}}_{l}^{k}={\bar{\mu }}_{f_{S}^{k}}(S^{PGO_{i}})/W_{l}$, and ${\underline{p}}_{l}^{k}={\underline{\mu }}_{f_{S}^{k}}(S^{PGO_{i}})/W_{l}$, in which $W_{l}=\sum_{k=1}^{L}{\bar{\mu }}_{f_{S}^{k}}(S^{PGO_{i}})+\sum_{k=L+1}^{M}{\underline{\mu }}_{f_{S}^{k}}(S^{PGO_{i}})$. The farthest right point of the interval type-2 set is defined as:

(14) $$y_{r}^{i}=\frac{\sum_{k=1}^{R}{\bar{\mu }}_{f_{s}^{k}}(S^{PGO_{i}})\alpha_{r}^{k}+\sum_{k=R+1}^{M}{\underline{\mu }}_{f_{s}^{k}}(S^{PGO_{i}})\alpha_{r}^{k}}{\sum_{k=1}^{R}{\bar{\mu }}_{f_{s}^{k}}(S^{PGO_{i}})+\sum_{k=R+1}^{M}{\underline{\mu }}_{f_{s}^{k}}(S^{PGO_{i}})}=\sum_{k=1}^{R}{\bar{p}}_{r}^{k}\alpha_{r}^{k}+\sum_{k=R+1}^{M}{\underline{p}}_{r}^{k}\alpha_{r}^{k}=[\begin{array}{cc} {\bar{\alpha }}_{r}^{T} & {\underline{\alpha }}_{r}^{T} \end{array}]\left[\begin{array}{c} {\bar{p}}_{r}\\ {\underline{p}}_{r} \end{array}\right]=\alpha_{r}^{T}\xi_{r},$$

where $\alpha_{r}^{k}$ is the farthest right point of $\alpha_{f_{u}}^{k}$, ${\bar{p}}_{r}^{k}={\bar{\mu }}_{f_{S}^{k}}(S^{PGO_{i}})/W_{r}$, and ${\underline{p}}_{r}^{k}={\underline{\mu }}_{f_{S}^{k}}(S^{PGO_{i}})/W_{r}$, in which $W_{r}=\sum_{k=1}^{R}{\bar{\mu }}_{f_{S}^{k}}(S^{PGO_{i}})+\sum_{k=R+1}^{M}{\underline{\mu }}_{f_{S}^{k}}(S^{PGO_{i}})$. As the PGOS is excited by the pulse-width modulation signal, the output of the interval type-2 fuzzy sliding pulse-width modulation controller, shown in Equation (12), has to be transferred by the “Pulse-Width Modulation Gen. Function”, which is:

(15) $$u^{PWM_{i}}=\left\{\begin{array}{l} u^{PGO_{i}}\cdot T^{PWM_{i}}\cdot 100\%\;\mathrm{up}{,\;\mathrm{if}\;u}^{PGO_{i}}\;>\;0\\ \left|u^{PGO_{i}}\right|\cdot T^{PWM_{i}}\cdot 100\%\;\mathrm{down}{,\;\mathrm{if}\;u}^{PGO_{i}}<0\\ 0\;\mathrm{stop},\;{\mathrm{if}\;u}^{PGO_{i}}=0\; \end{array}\right.,$$

where $u^{PWM_{i}}$ is the duty cycle to the *i*th pneumatic actuator for the PGOS. Here, the interval type-2 fuzzy sliding pulse-width modulation control provides the pulse-width modulation command to the pneumatic actuator at a sampling frequency of 50 Hz. We can find that if $u^{PGO_{i}}>0$, the pneumatic actuator moves up, while if $u^{PGO_{i}}<0$, the pneumatic actuator stops. Figure 14b illustrates the overall control block for the PGOS, in which four independent interval type-2 fuzzy sliding pulse-width modulation controllers are applied for the four pneumatic actuators.

### 4.3. Design of an Interval Type-2 Fuzzy Sliding Controller for the PBWSS

**Figure 15:**
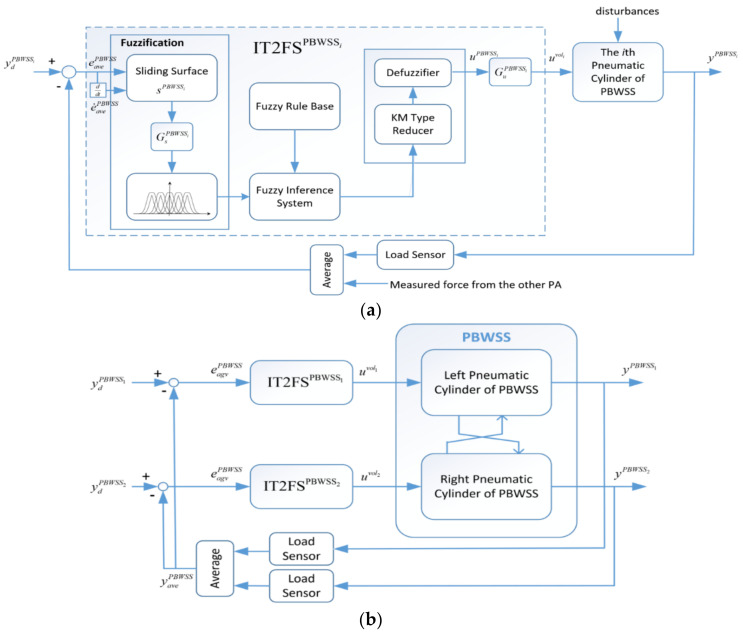
Block diagram of the IT2FSC for the PBWSS (**a**) the *i*th IT2FSC; (**b**) the overall control block for the PBWSS.

The PBWSS’s motion is regulated by two pressure control proportional valves. The pressure control proportional valve is more expensive than the on-off valve, but it allows outputting an accurate pressure force depending on an input voltage; hence, a controller can be easily and straightforwardly designed to produce precise force for the PBWSS. The PBWSS has to compensate uncertainties and disturbances, and it shall provide reliable unloading force for a patient who may exert extra force (i.e., his/her body weight). To overcome the above-mentioned difficulties, this study designed an IT2FSC which uses a sliding surface as an input variable to formulate a voltage output, and the voltage enables the force through the pressure control proportional valve. Figure 15a shows a block diagram of the force control with the IT2FSC, denoted as ${\mathrm{IT}2\mathrm{FS}}^{{\mathrm{PBWSS}}_{i}}$, for the *i*th pneumatic actuator. Here, the inference from the other pneumatic actuator is considered as a disturbance, and the feedback force is defined as an average of the two external forces imposing on the two pneumatic actuators. $G_{s}^{PBWSS_{i}}$ and $G_{u}^{PBWSS_{i}}$ are the scalar factors for the input and output of the ${\mathrm{IT}2\mathrm{FS}}^{{\mathrm{PBWSS}}_{i}}$, respectively. Figure 15b illustrates the overall control block for the PBWSS, in which two independent IT2FSCs are, respectively, applied for two pneumatic actuators. $y^{PBWSS_{1}}$ is the output force of the right linear actuator, and $y^{PBWSS_{2}}$ is the output force of the left linear actuator. The *i*th IT2FSC ${\mathrm{IT}2\mathrm{FS}}^{{\mathrm{PBWSS}}_{i}}$ outputs the voltage $u^{vol_{i}}$ for the pneumatic actuator. The input for both ${\mathrm{IT}2\mathrm{FS}}^{{\mathrm{PBWSS}}_{i}}$ (i=1,2) is defined as the error $e_{avg}^{PBWSS}$, which is:

(16) $$e_{avg}^{PBWSS}=y_{d}^{PBWSS}-\frac{1}{2}(y^{PBWSS_{1}}+y^{{\mathrm{PBWSS}}_{2}}),$$

$y_{d}^{PBWSS}$ is the reference input. Define $y_{avg}^{PBWSS}=(1/2)(y^{PBWSS_{1}}+y^{PBWSS_{1}})$ as the average of the two outputs, and let ${\dot{y}}_{avg}^{PBWSS}$ be the its derivative and ${\dot{y}}_{d}^{PBWSS}$ be the derivative of the reference input. Then, the sliding surface can be defined as:

(17) $$S^{PBWSS_{i}}(t)=\lambda_{1}^{i}e^{PBWSS_{i}}(t)+\lambda_{2}^{i}{\dot{e}}^{PBWSS_{i}}(t),$$

where $e^{PBWSS_{i}}(t)=y_{avg}^{PBWSS}(t)-y_{d}^{PBWSS}(t)$, ${\dot{e}}^{PBWSS_{i}}(t)={\dot{y}}_{avg}^{PBWSS}(t)-{\dot{y}}_{d}^{PBWSS}(t)$, and $c_{j}^{i}$ are specified such that $\sum_{j=1}^{2}c_{j}^{i}\lambda_{i}^{j-1}$ is a Hurwitz polynomial and $\lambda_{i}^{j-1}$ is a Laplace operator. $\lambda_{i}^{j-1}$ can be a different value between the two ${\mathrm{IT}2\mathrm{FS}}^{{\mathrm{PBWSS}}_{i}}$. The lth fuzzy rule for the ${\mathrm{IT}2\mathrm{FS}}^{PBWSS_{i}}$ is:

(18) $$R^{l}:if\;S^{PBWSS_{i}}\;\mathrm{is}\;f_{s}^{l}\;then\;u^{PBWSS_{i}}\;\mathrm{is}\;f_{u}^{l}\;,\;(l=1,\ldots ,M),$$

where $f_{s}^{l}$ is an interval type-2 fuzzy set and $f_{u}^{l}$ is an interval type-2 singleton fuzzy set. Please note that $f_{s}^{l}$ and $f_{u}^{l}$ can be different fuzzy sets between the two IT2FSCs. The output of the IT2FSC calculated by singleton fuzzification, the product inference and the center-average defuzzification is given as:

(19) $$u^{PBWSS_{i}}(S^{PBWSS_{i}},\alpha )=\frac{y_{l}^{i}+y_{r}^{i}}{2}=\frac{1}{2}\left[\alpha_{l}^{T}\alpha_{r}^{T}\right]\left[\begin{array}{l} \xi_{l}\\ \xi_{r} \end{array}\right]=\alpha^{T}\xi ,$$

where $u^{PBWSS_{i}}$ is the voltage output of the *i*th IT2FSC, $y_{l}^{i}$ and $y_{r}^{i}$, respectively, represent the farthest left and the farthest right points of the interval type-2 fuzzy set for the *i*th IT2FSC. $\alpha^{T}=\left[\alpha_{1},\alpha_{2},\cdots ,\alpha_{2M}\right]$ is a weight vector. The KM algorithm is used for the type reducer. The farthest left point for the interval type-2 fuzzy set is defined as:

(20) $$y_{l}^{i}=\frac{\sum_{k=1}^{L}{\bar{\mu }}_{f_{S}^{k}}(S^{PBWSS_{i}})\alpha_{l}^{k}+\sum_{k=L+1}^{M}{\underline{\mu }}_{f_{S}^{k}}(S^{PBWSS_{i}})\alpha_{l}^{k}}{\sum_{k=1}^{L}{\bar{\mu }}_{f_{S}^{k}}(S^{PBWSS_{i}})+\sum_{k=L+1}^{M}{\underline{\mu }}_{f_{S}^{k}}(S^{PBWSS_{i}})}=\sum_{k=1}^{L}{\bar{p}}_{l}^{k}\alpha_{l}^{k}+\sum_{k=L+1}^{M}{\underline{p}}_{l}^{k}\alpha_{l}^{k}=[\begin{array}{cc} {\bar{\alpha }}_{l}^{T} & {\underline{\alpha }}_{l}^{T} \end{array}]\left[\begin{array}{c} {\bar{p}}_{l}\\ {\underline{p}}_{l} \end{array}\right]=\alpha_{l}^{T}\xi_{l},$$

where ${\bar{\mu }}_{f_{S}^{k}}$ and ${\underline{\mu }}_{f_{S}^{k}}$, respectively, represent the upper and lower degrees of the membership function, $\alpha_{l}^{k}$ is the farthest left point of $\alpha_{f_{u}}^{k}$, ${\bar{p}}_{l}^{k}={\bar{\mu }}_{f_{S}^{k}}(S^{PBWSS_{i}})/W_{l}$, and ${\underline{p}}_{l}^{k}={\underline{\mu }}_{f_{S}^{k}}(S^{PBWSS_{i}})/W_{l}$, in which $W_{l}=\sum_{k=1}^{L}{\bar{\mu }}_{f_{S}^{k}}(S^{PBWSS_{i}})+\sum_{k=L+1}^{M}{\underline{\mu }}_{f_{S}^{k}}(S^{PBWSS_{i}})$. The farthest right point of the interval type-2 set is defined as:

(21) $$y_{r}^{i}=\frac{\sum_{k=1}^{R}{\bar{\mu }}_{f_{s}^{k}}(S^{PBWSS_{i}})\alpha_{r}^{k}+\sum_{k=R+1}^{M}{\underline{\mu }}_{f_{s}^{k}}(S^{PBWSS_{i}})\alpha_{r}^{k}}{\sum_{k=1}^{R}{\bar{\mu }}_{f_{s}^{k}}(S^{PBWSS_{i}})+\sum_{k=R+1}^{M}{\underline{\mu }}_{f_{s}^{k}}(S^{PBWSS_{i}})}=\sum_{k=1}^{R}{\bar{p}}_{r}^{k}\alpha_{r}^{k}+\sum_{k=R+1}^{M}{\underline{p}}_{r}^{k}\alpha_{r}^{k}=[\begin{array}{cc} {\bar{\alpha }}_{r}^{T} & {\underline{\alpha }}_{r}^{T} \end{array}]\left[\begin{array}{c} {\bar{p}}_{r}\\ {\underline{p}}_{r} \end{array}\right]=\alpha_{r}^{T}\xi_{r},$$

where $\alpha_{r}^{k}$ is the farthest right point of $\alpha_{f_{u}}^{k}$, ${\bar{p}}_{r}^{k}={\bar{\mu }}_{f_{S}^{k}}(S^{PBWSS_{i}})/W_{r}$ and ${\underline{p}}_{r}^{k}={\underline{\mu }}_{f_{S}^{k}}(S^{PBWSS_{i}})/W_{r}$, in which $W_{r}=\sum_{k=1}^{R}{\bar{\mu }}_{f_{S}^{k}}(S^{PBWSS_{i}})+\sum_{k=R+1}^{M}{\underline{\mu }}_{f_{S}^{k}}(S^{PBWSS_{i}})$.

## 5. Experiments Results and Discussion

The goal of the experiments in this paper was to evaluate the feasibility of the PRPGTS regulated by the interval type-2 fuzzy sliding pulse-width modulation controllers and IT2FC. Three experiments are reported in this section. In Section 5.1, an experiment used to verify the motion control of the PGOS as a gait training cycle is given. Two experiments are presented in Section 5.2 and Section 5.3 to show the effectiveness of the PBWSS; the first experiment presents static bodyweight unloading force control, and the second shows the dynamic bodyweight unloading force control of the PBWSS. The static bodyweight unloading force control aims to examine the bodyweight reduction function while the PGOS powers off, and the purpose of the dynamic bodyweight unloading control is to verify the bodyweight reduction function when the is PGOS enabled. In the experiments, the subject tested on the PRPGTS was a 172 cm tall and 68 kg healthy male, and the interval type-2 fuzzy sliding pulse-width modulation controllers and the IT2FSC were developed in the LabVIEW environment and implemented in the FPGA-based embedded system to allow a real-time control. This study uses an output feedback control algorithm. To reduce the cost of PRPGTS, the angular velocity and the force change rate are calculated using a numerical difference operation, differentiating the angle and force for time numerically. Since the angular velocity and the force change rate are subject to disturbances from the numerical difference operation, a digital filter expressed as follows is, hence, introduced to solve this problem:

(22) $$y_{out}(i)=-0.047y_{out}(i-1)+0.524[y_{in}(i)+y_{in}(i-1)],$$

where $y_{out}(t)$ stands for the filter’s output signal, while $y_{in}(t)$ is the sensor’s measured data.

The pneumatic force has to be slow and smooth to provide comfort and safe control for a subject. For this purpose, we designed a fifth-order polynomial continuous function as the tracking trajectory for the PRPGTS in these experiments. At first, the reference signals $y_{d}^{PGO_{i}}(t)$ and $y_{d}^{PBWSS_{i}}(t)$ are segmented as sequences $\left\{y_{d}^{PGO_{i}}(t_{f_{0}})\;y_{d}^{PGO_{i}}(t_{f_{1}})\;y_{d}^{PGO_{i}}(t_{f_{2}})\;y_{d}^{PGO_{i}}(t_{f_{3}})\;\ldots \;\right\}$ and $\left\{y_{d}^{PBWSS_{i}}(t_{f_{0}})\;y_{d}^{PBWSS_{i}}(t_{f_{1}})\;y_{d}^{PBWSS_{i}}(t_{f_{2}})\;y_{d}^{PBWSS_{i}}(t_{f_{3}})\;\ldots \;\right\}$, respectively, and the reference signals $y_{d}^{PGO_{i}}(t)$ and $y_{d}^{PBWSS_{i}}(t)$ during the time interval of $\left[t_{f_{i-1}}\;t_{f_{i}}],(i=1,2,\ldots \right)$ are formed by the fifth-order polynomial continuous function with the following conditions: 1. the initial variations (i.e., $y_{d}^{}(t_{1}=0)$, ${\dot{y}}_{d}^{}(t_{1}=0)$ and ${\ddot{y}}_{d}^{}(t_{1}=0)$) are zero, and 2. the reached variations (i.e., ${\dot{y}}_{d}^{}(t_{1}=t_{f})$ and ${\ddot{y}}_{d}^{}(t_{1}=t_{f})$) are zero. The fifth-order polynomial continuous function can be expressed as:

(23) $$y_{d}^{}(t_{1})=\left\{\begin{array}{c} h\left[10{\left(\frac{t_{1}}{t_{f_{i}}}\right)}^{3}-15{\left(\frac{t_{1}}{t_{f_{i}}}\right)}^{4}+6{\left(\frac{t_{1}}{t_{f_{i}}}\right)}^{5}\right],\;0\leq t_{1}<(t_{f_{i}}-t_{f_{i-1}})\\ \begin{array}{c} h\;,\;t_{1}\geq (t_{f_{i}}-t_{f_{i-1}}) \end{array} \end{array}\right.,i=1,2,3,\ldots ,$$

where h is a desired control output at each time interval, $\left(t_{f_{i}}-t_{f_{i-1}}\right)$ denotes a time duration, $t_{1}$ is set to zero at the beginning of each time interval, and the sampling time is chosen as 0.005 s.

### 5.1. Control of the Motion for the PGOS Using Interval Type-2 Fuzzy Sliding Pulse-Width Modulation Controllers

In this experiment, a healthy subject 172-cm tall and of 68-kg weight wore the PGOS for ten seconds, and the PGOS was enabled and regulated by four individual interval type-2 fuzzy sliding pulse-width modulation controllers. The design steps of the PGOS with the interval type-2 fuzzy sliding pulse-width modulation controller were as follows:

**Step** **1:** The time scalar κ of the full gait cycle is set to 2 for the ten-second examination. The reference translational trajectories, i.e.,$y_{d}^{PGO_{1}}$, $y_{d}^{PGO_{2}}$, $y_{d}^{PGO_{3}}$ and $y_{d}^{PGO_{4}}$, for the joints of the lower limb exoskeleton are calculated by Equations (7) and (8).
**Step** **2:** Power on the pneumatic postural support system.
**Step** **3:** The interval type-2 fuzzy sliding pulse-width modulation controller $u^{PWM_{i}}(i=1,\ldots ,4)$ is designed according to Equations (12) and (15) with the parameters given in Table 3.

**Figure 16:**
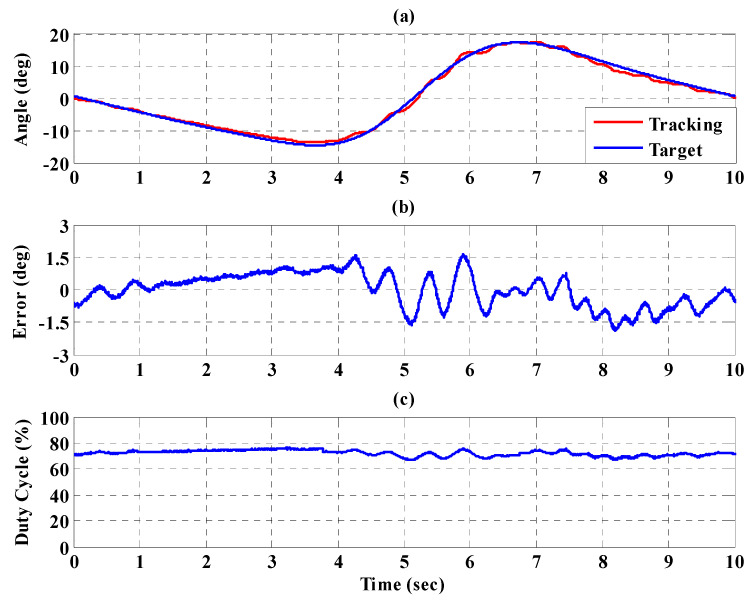
Tracking response of the interval type-2 fuzzy sliding pulse-width modulation controller on the right hip for the PGOS. (**a**) output response; (**b**) tracking error; (**c**) pulse-width modulation control signal.

**Figure 17:**
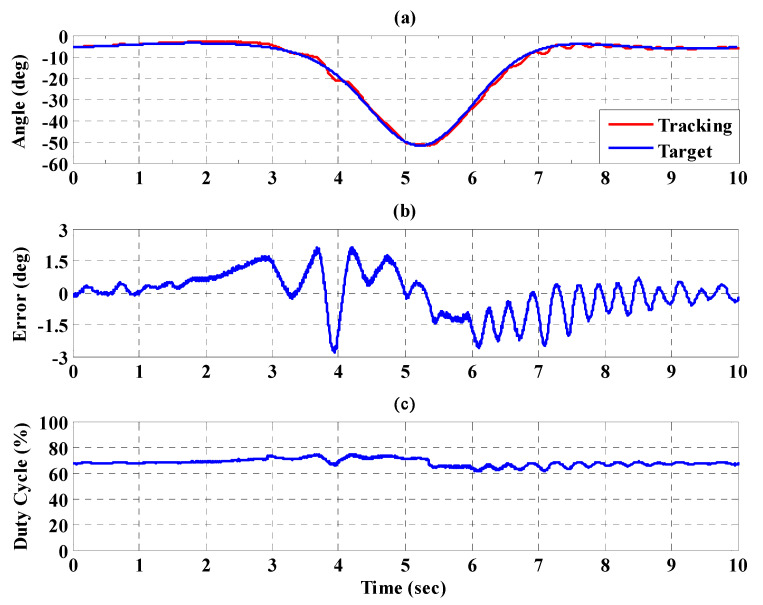
Tracking response of the interval type-2 fuzzy sliding pulse-width modulation controller on the right knee for the PGOS. (**a**) output response; (**b**) tracking error; (**c**) pulse-width modulation control signal.

**Figure 18:**
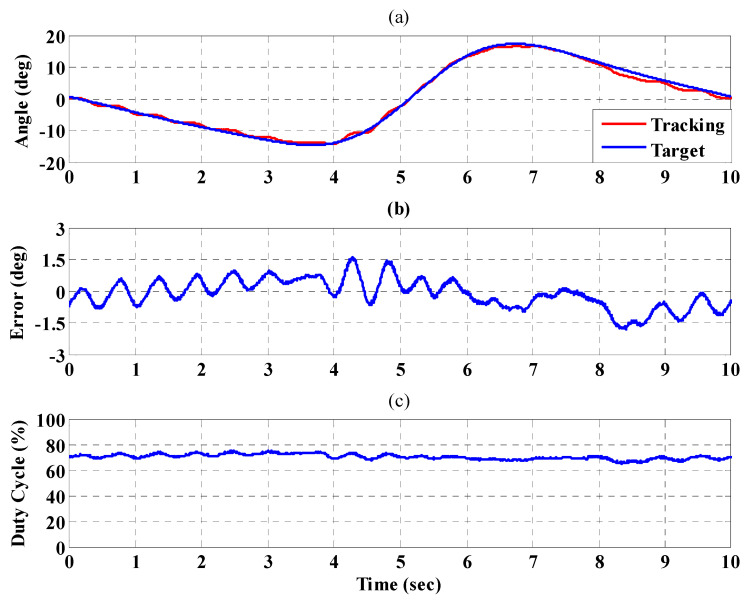
Tracking response of the interval type-2 fuzzy sliding pulse-width modulation controller on the left hip for the PGOS. (**a**) output response; (**b**) tracking error; (**c**) pulse-width modulation control signal.

**Figure 19:**
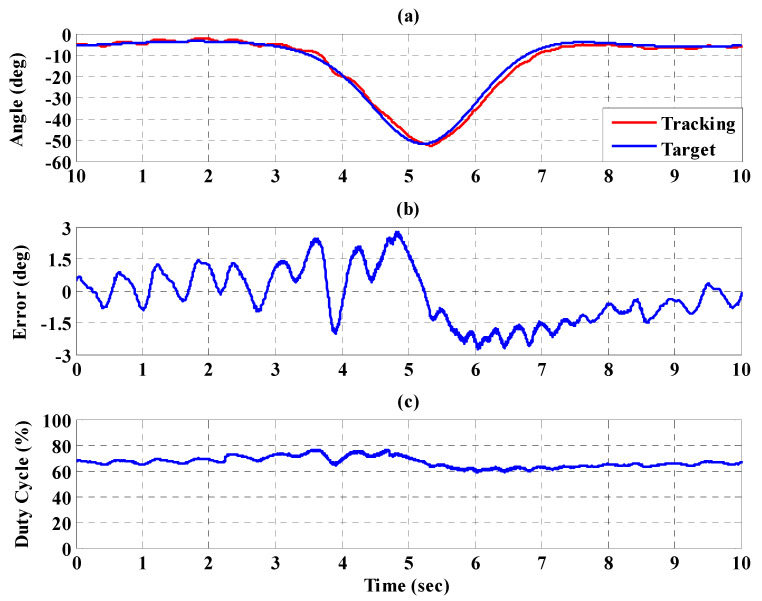
Tracking response of the interval type-2 fuzzy sliding pulse-width modulation controller on the left knee for the PGOS. (**a**) output response; (**b**) tracking error; (**c**) pulse-width modulation control signal.

Figure 16a,b, respectively, show the trajectory tracking response and the tracking error for the right hip when using the interval type-2 fuzzy sliding pulse-width modulation controller for the PGOS. We can see that the absolute maximum of the tracking error is less than 1.6 degrees during the whole gait training process. Figure 16c shows the pulse-width modulation control signal of the interval type-2 fuzzy sliding pulse-width modulation controller for the right hip. Figure 17a,b, respectively, show the trajectory tracking response and the tracking error for the right knee. The absolute maximum of the tracking error is less than 2.8 degrees. Figure 17c shows the pulse-width modulation control signal of the interval type-2 fuzzy sliding pulse-width modulation controller for the right knee. Figure 18a,b, respectively, show the trajectory tracking response and the tracking error for the left hip using the interval type-2 fuzzy sliding pulse-width modulation controller for the PGOS. The absolute maximum of the tracking error is less than 1.6 degrees during gait training. Figure 18c shows the pulse-width modulation control signal of the interval type-2 fuzzy sliding pulse-width modulation controller for the left hip. Figure 19a,b, respectively, show the trajectory tracking response and the tracking error for the left knee. The absolute maximum of the tracking error is less than 2.9 degrees. Figure 19c shows the pulse-width modulation control signal of the interval type-2 fuzzy sliding pulse-width modulation controller for the left knee. As reported by [42], the motion of the lower limb is not identical between different people or even for one person at different times, so the 10% motion error for the hip and the knee during gait training is acceptable in clinical practice. Clearly, the results show that the motion error in PGOS with interval type-2 fuzzy sliding pulse-width modulation controller is less than 10%.

### 5.2. Static Bodyweight Unloading Force Control for the PBWSS

The procedure for the static bodyweight unloading force control is described as following: before enabling the PBWSS, a 172 cm tall and 68 kg weight subject wears the PGO and turns the PGOS off. The design steps of the PBWSS with the IT2FSC are as follows:

**Step** **1:** The targeted weight reduction for the subject is set to 20% (13.6 kg weight loss), 30% (20.4 kg weight loss), and 40% (27.2 kg weight loss) in this experiment. The load cell directly senses the weight of the subject and sends it back to the IT2FSC, so that the reference inputs $y_{d}^{PWBSS_{1}}$ and $y_{d}^{PWBSS_{2}}$ can be chosen as the targeted weight; that is $y_{d}^{PWBSS_{1}}=y_{d}^{PWBSS_{2}}=54.4$ kg for the experiment with a 20% weight reduction, $y_{d}^{PWBSS_{1}}=y_{d}^{PWBSS_{2}}=47.6$ kg for the experiment with 30% weight reduction, $y_{d}^{PWBSS_{1}}=y_{d}^{PWBSS_{2}}=40.8$ kg for the experiment with 40% weight reduction. The reference inputs $y_{d}^{PWBSS_{1}}$ and $y_{d}^{PWBSS_{2}}$ are described as the fifth-order polynomial continuous function to ensure the PBWSS moves smoothly, stably, and safely.
**Step** **2:** Power on the pneumatic postural support system.
**Step** **3:** The controller IT2FSC $u^{vol_{i}}(i=1,2)$ is designed according to Equation (19), with the parameters given in Table 4.

**Figure 20:**
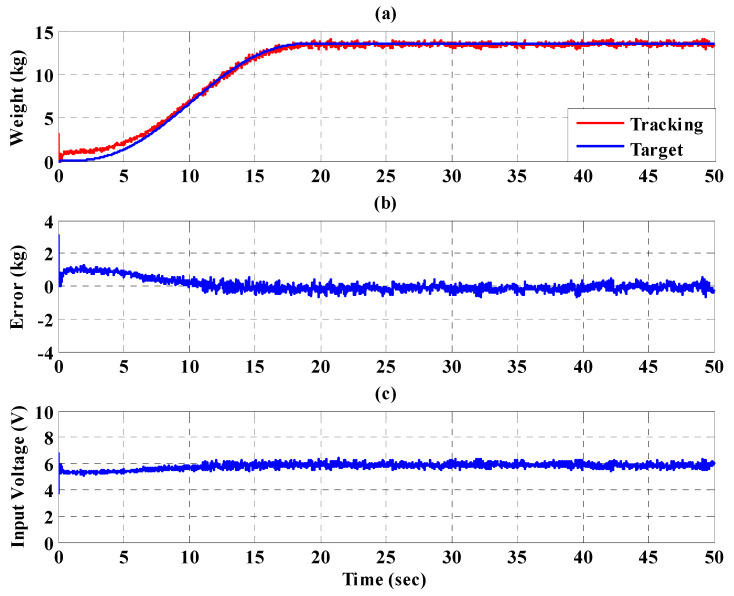
Experimental results for the PBWSS with a static bodyweight unloading force of 20% (13.6 kg) in 50 s. (**a**) Tracking trajectory; (**b**) tracking error; (**c**) control voltage.

**Figure 21:**
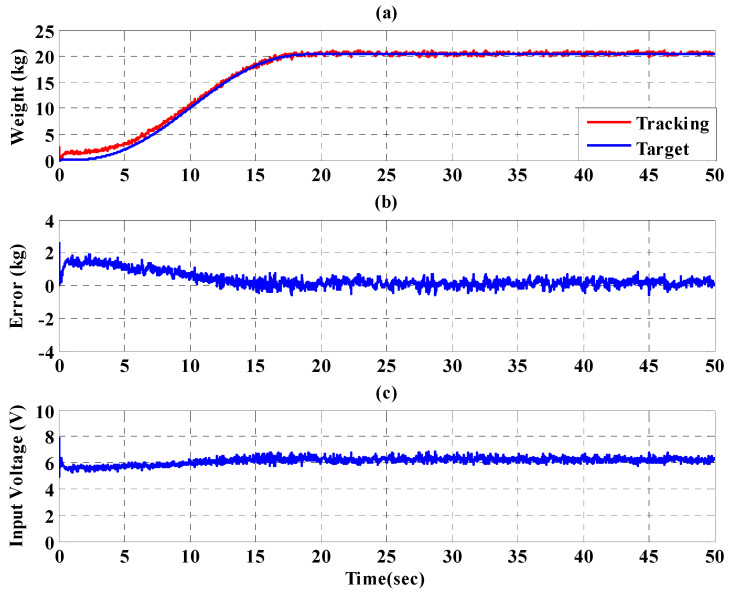
Experimental results for the PBWSS with a static bodyweight unloading force of 30% (20.4 kg) in 50 s. (**a**) Tracking trajectory; (**b**) tracking error; (**c**) control voltage.

**Figure 22:**
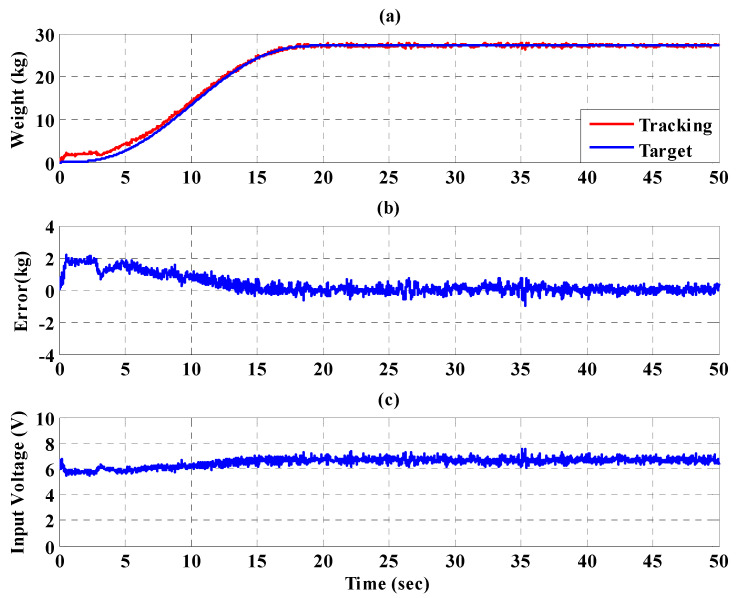
Experimental results for the PBWSS with a static bodyweight unloading force of 40% (27.2 kg) in 50 s. (**a**) Tracking trajectory; (**b**) tracking error; (**c**) control voltage.

To increase safety for the subject, the IT2FSC is designed to output a gentle and smooth control force to the PBWSS in 50 s. Figure 20, Figure 21 and Figure 22, respectively, show the experimental results for the 10%, 20%, and 30% static bodyweight unloading force controls for the PBWSS with the IT2FSCFigure 20a, Figure 21a, and Figure 22a, respectively, show that 10%, 20%, and 30% static unloading force reductions can be achieved in about 20 s. The tracking errors for the static bodyweight unloading force are less than ±2 kg after 20 s, as shown in Figure 20b, Figure 21b, and Figure 22b. Figure 20c, Figure 21c, and Figure 22c show the output control voltage. The experimental results show that the PBWSS with the IT2FSC effectively realized the weight reduction for the subject.

### 5.3. Dynamic Bodyweight Unloading Force Control for the PBWSS

The procedure of the dynamic bodyweight unloading force control is described as follows: A 172 cm tall and 68 kg weight subject wears the PGO. Then, they turn the PGOS on and enable the PBWSS. The design steps of the PBWSS with the IT2FSC are as follows:

**Step** **1:** The targeted weight reduction for the subject was set to 20% (13.6 kg weight loss), 30% (20.4 kg weight loss), and 40% (27.2 kg weight loss) in this experiment. The load cell directly senses the weight of the subject and sends it back to the IT2FSC, so the reference inputs $y_{d}^{PWBSS_{1}}$ and $y_{d}^{PWBSS_{2}}$ are defined as the targeted weight; that is $y_{d}^{PWBSS_{1}}=y_{d}^{PWBSS_{2}}=54.4$ kg for the experiment with a 20% weight reduction,$y_{d}^{PWBSS_{1}}=y_{d}^{PWBSS_{2}}=47.6$ kg for the experiment with 30% weight reduction, and $y_{d}^{PWBSS_{1}}=y_{d}^{PWBSS_{2}}=40.8$ kg for the experiment with 40% weight reduction. The reference inputs $y_{d}^{PWBSS_{1}}$ and $y_{d}^{PWBSS_{2}}$ are described as the fifth-order polynomial continuous function to ensure the PBWSS moves smoothly, stably, and safely.
**Step** **2:** Power on the pneumatic postural support system.
**Step** **3:** The controller IT2FSC $u^{vol_{i}}(i=1,2)$ was designed according to Equation (19), with the parameters given in Table 5.
**Step** **4:** The procedure of the PGOS control is identical to experiment 1. The interval type-2 fuzzy sliding pulse-width modulation controllers $u^{PWM_{i}}(i=1,\ldots ,4)$ were designed according to Equations (12) and (15), with the parameters given in Table 3.

**Figure 23:**
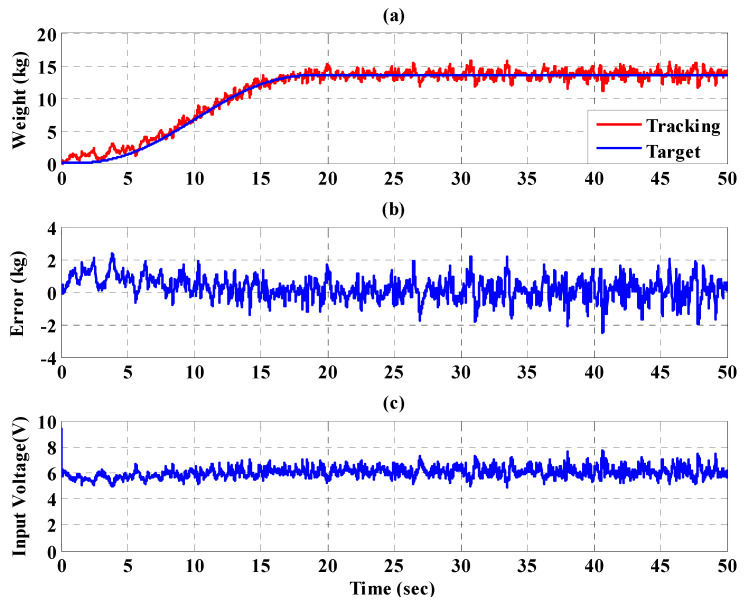
Experimental results for the PBWSS with a dynamic bodyweight unloading force of 20% (13.6 kg) in 50 s. (**a**) Tracking trajectory; (**b**) tracking error; (**c**) control voltage.

**Figure 24:**
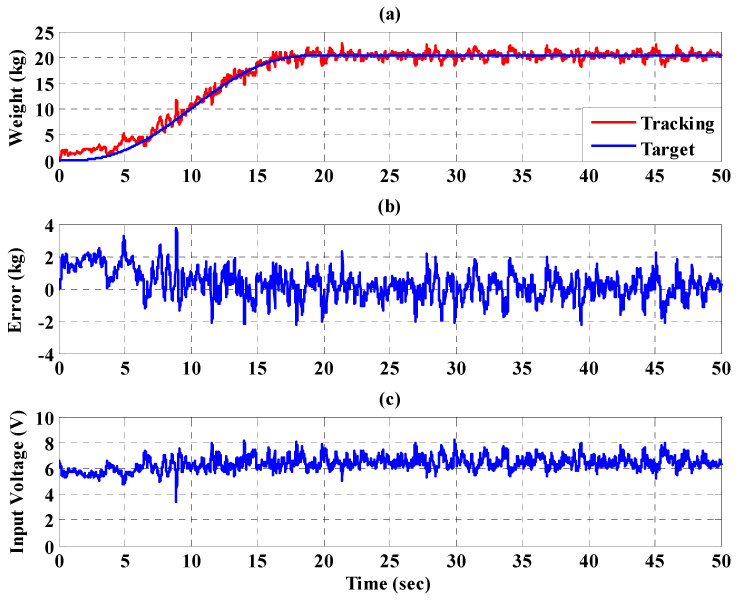
Experimental results for the PBWSS with a dynamic bodyweight unloading force of 30% (20.4 kg) in 50 s. (**a**) Tracking trajectory; (**b**) tracking error; (**c**) control voltage.

**Figure 25:**
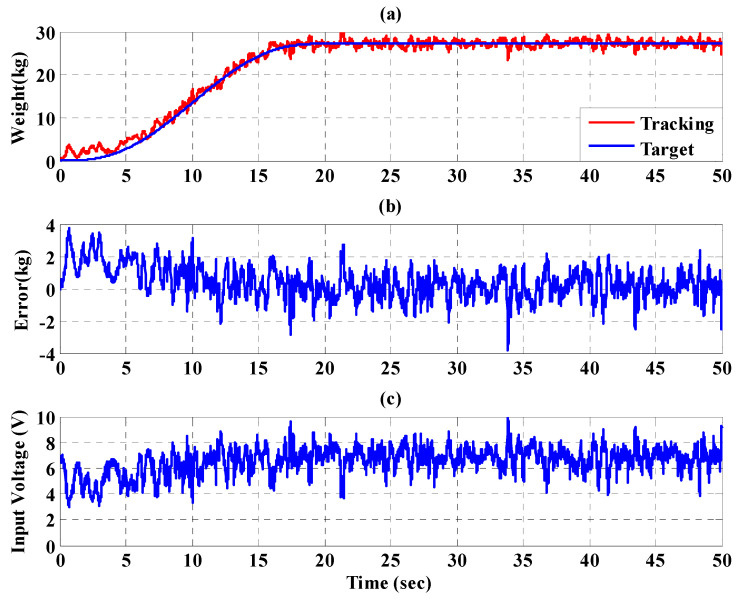
Experimental results for the PBWSS with a dynamic bodyweight unloading force of 40% (27.2 kg) in 50 s. (**a**) Tracking trajectory; (**b**) tracking error; (**c**) control voltage.

**Figure 26:**
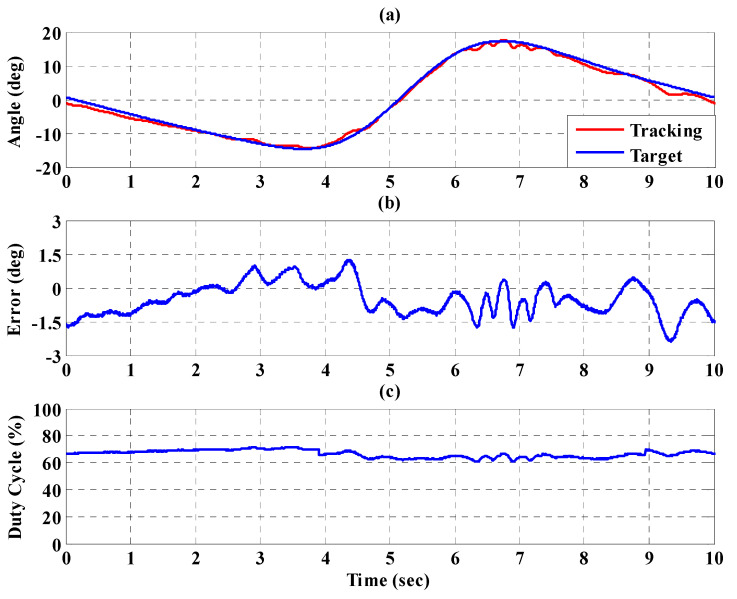
Tracking response of the interval type-2 fuzzy sliding pulse-width modulation controller on the right hip for the PGOS. (**a**) Output response; (**b**) tracking error; (**c**) pulse-width modulation control signal.

**Figure 27:**
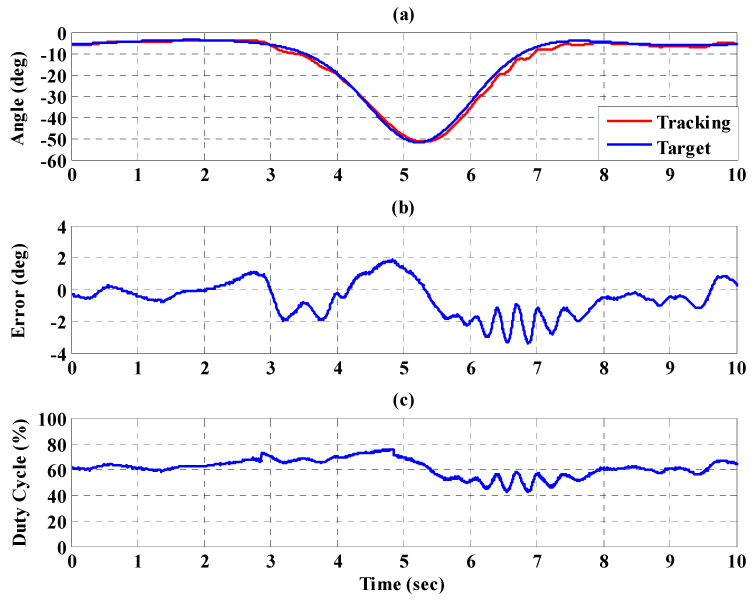
Tracking response of the interval type-2 fuzzy sliding pulse-width modulation controller on the right knee for the PGOS. (**a**) Output response; (**b**) tracking error; (**c**) pulse-width modulation control signal.

**Figure 28:**
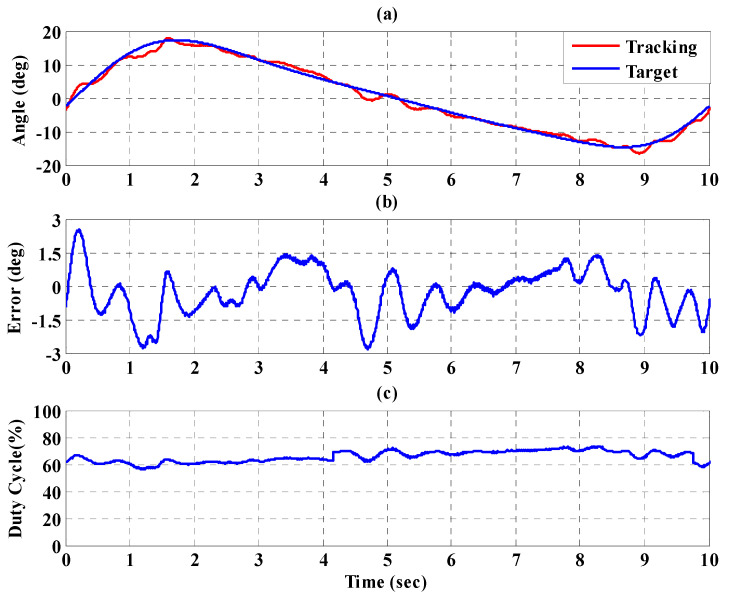
Tracking response of the interval type-2 fuzzy sliding pulse-width modulation controller on the left hip for the PGOS. (**a**) Output response; (**b**) tracking error; (**c**) pulse-width modulation control signal.

**Figure 29:**
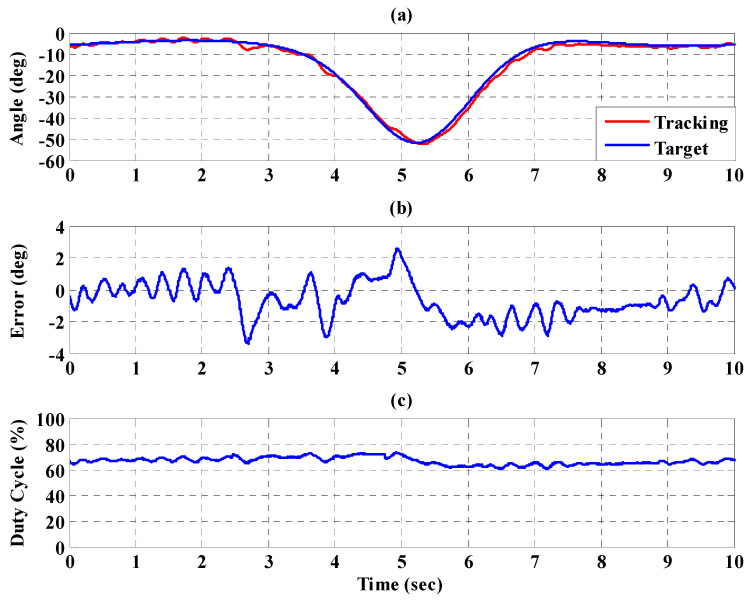
Tracking response of the interval type-2 fuzzy sliding pulse-width modulation controller on the left knee for the PGOS. (**a**) Output response; (**b**) tracking error; (**c**) pulse-width modulation control signal.

Figure 23, Figure 24 and Figure 25, respectively, show the experimental results for the 10%, 20%, and 30% static bodyweight unloading force control for the PBWSS with the IT2FSC. Figure 23a, Figure 24a, and Figure 25a, respectively, show that 10%, 20%, and 30% dynamic unloading force reductions can be achieved in about 20 s. The tracking errors for the dynamic bodyweight unloading force are limited to around ±2 kg after 20 s, as shown in Figure 23b, Figure 24b, and Figure 25b. Figure 23c, Figure 24c, and Figure 25c show the output control voltage. The experimental results show that the PBWSS with the IT2FSC effectively realized the weight reduction for the patient. Figure 26a,b, respectively, show the trajectory tracking response and the tracking error for the right hip when using the interval type-2 fuzzy sliding pulse-width modulation controller for the PGO. We can see that the absolute maximum of the tracking error was less than 1.8 degrees during the whole gait training process. Figure 26c shows the pulse-width modulation signal of the interval type-2 fuzzy sliding pulse-width modulation controller for the right hip. Figure 27a,b, respectively, show the trajectory tracking response and the tracking error for the left knee. The absolute maximum of the tracking error was less than 3.6 degrees during gait training. Figure 27c shows the pulse-width modulation control signal of the interval type-2 fuzzy sliding pulse-width modulation controller for the right knee. Figure 28a,b, respectively, show the trajectory tracking response and the tracking error for the left hip when using the interval type-2 fuzzy sliding pulse-width modulation controller for the PGO. The absolute maximum of the tracking error was less than 2.8 degrees during gait training. Figure 28c shows the pulse-width modulation control signal of the interval type-2 fuzzy sliding pulse-width modulation controller for the left hip. Figure 29a,b, respectively, show the trajectory tracking response and the tracking error for the left knee. The absolute maximum of the tracking error was less than 3.6 degrees during gait training. Figure 29c shows the pulse-width modulation control signal of the interval type-2 fuzzy sliding pulse-width modulation controller for the left knee. The results show that the PGOS with interval type-2 fuzzy sliding pulse-width modulation controllers also have a performance with a better than 10% motion error.

## 6. Conclusions

We manufactured a prototype of the pneumatic-driven passive robotic gait training system (PPRGTS) for patients who suffer from weakened lower limbs and designed two types of IT2FSC to overcome system uncertainties and external loading. The PRGTS is comprised of three subsystems: the PBWSS enables the function of reducing body-weight loading from the subject, the PPSS provides the function of balancing the subject’s body, and the PBWSS has the function of driving the subject’s legs following a given gait training cycle. In the experiments, the static bodyweight unloading force control and the dynamic bodyweight unloading force control showed that the PBWSS successfully achieved the weight reduction for the subject; a 172 cm tall and 68 kg healthy male. We found that the absolute maximum of the tracking error for all joints of the PGOS can be reduced to the desired value (10% of the targeted degree) during the whole gait training process after using the IT2FSPWMC for the PGOS. The feasibility of the PPRGTS was demonstrated because it had a small trajectory tracking error during gait training and provided a stable weight reduction function. In addition, because the pneumatic driver is a soft actuator, it provided passive safety for the targets in the experiments.

## 7. Patents

The pneumatic-driven robotic gait training system developed has obtained a Taiwanese invention patent and a US invention patent. These are (1) PNEUMATIC LOWER EXTREMITY GAIT REHABILITATION TRAINING SYSTEM, Taiwanese patent number I55556; (2) PNEUMATIC LOWER EXTREMITY GAIT REHABILITATION TRAINING SYSTEM, US patent number US 10,292,892 B2.

## Figures and Tables

**Table 1:** Components of the PGOS.

Components	Type	Part of the PGOS
Single-rod pneumatic cylinder	CHELIC SDA32–150	Hip joints for both legs
Single-rod pneumatic cylinder	CHELIC SDA32–100	Knee joints for both legs
Single-rod pneumatic cylinder	CHELIC SDA32–75	Ankle joints for both legs
Proportional pressure regulator	FESTO VPPM-6L-L-1-G18-0L6H-V1P-S1	Hip joints, knee joint, and ankle joints for both legs
Incremental encoders	ELCO E38F8-C4AR-2000 at the maximum frequency of 300 kHz	Joints of the PGOS

**Table 2:** Joint Denavit–Hartenberg Coordinate Parameters.

Joint i	$\theta_{i}/{}^{\circ}$	$d_{i}$ /mm	$a_{i}$ /mm	$\alpha_{i}/{}^{\circ}$	Joint Variable
1	$\theta_{1}$	0	$a_{1}$	0	$\theta_{1}$
2	$\theta_{2}$	0	$a_{2}$	0	$\theta_{2}$
3	$\theta_{3}$	0	$a_{3}$	0	$\theta_{3}$

**Table 3:** Control parameters of the interval type-2 fuzzy sliding pulse-width modulation controller for the PGOS.

**Membership function of** $S^{PGO_{i}}$	$M\left(S^{PGO_{i}}\right)=\left[\left(c_{1},c_{2},\sigma \right)\right]=\left[\begin{array}{ccc} \left(-22,-20,1.5\right) & \left(-16,-14,1.5\right) & \left(-7.7,-6.7,1.5\right)\\ \left(-4.6,-2.6,1.5\right) & \left(-1,1,1.5\right) & \left(2.1,4.5,1.5\right)\\ \left(6.4,8.7,1.5\right) & \left(13,15,1.5\right) & \left(20,22,1.5\right) \end{array}\right]$			
**Membership function of** $u^{PGO_{i}}$	$M\left(u^{PGO_{i}}\right)=\left[\left(u_{l},u_{r}\right)\right]=\left[\begin{array}{ccc} \left(0,0.1\right) & \left(0.13,0.14\right) & \left(0.16,0.23\right)\\ \left(0.29,0.38\right) & \left(0.55,0.6\right) & \left(0.75,0.81\right)\\ \left(0.86,0.88\right) & \left(0.89,0.95\right) & \left(0.98,1\right) \end{array}\right]$			
**Control parameters of joints**	$\lambda_{1}$	$\lambda_{2}$	$G_{s}^{PGO}$	$G_{u}^{PGO}$
**Right hip**	0.5	0.4	0.5	1.2
**Right knee**	0.8	0.9	0.7	1.2
**Left hip**	0.7	1	0.5	1.1
**Left knee**	0.5	0.2	0.4	1.3

**Table 4:** The used parameters of the PBWSS with the IT2FSC for the static unloading force control.

**Membership function of** $F^{i}$	$M\left(F^{i}\right)=\left[\left(c_{1},c_{2},\sigma \right)\right]=\left[\begin{array}{ccc} \left(-22,-20,1.5\right) & \left(-13,-11,1.5\right) & \left(-8.7,-6.7,1.5\right)\\ \left(-4.6,-2.6,1.5\right) & \left(-1,1,1.5\right) & \left(2.1,4.5,1.5\right)\\ \left(6.4,8.7,1.5\right) & \left(13,15,1.5\right) & \left(20,22,1.5\right) \end{array}\right]$			
**Membership function of** $y^{i}$	$M\left(y^{i}\right)=\left[\left(y_{l},y_{r}\right)\right]=\left[\begin{array}{ccc} \left(10,9.5\right) & \left(9.2,9\right) & \left(8.5,8\right)\\ \left(7.5,7\right) & \left(6.6,6\right) & \left(5.5,5\right)\\ \left(3.5,3\right) & \left(2.5,2\right) & \left(1.5,0\right) \end{array}\right]$			
**Control parameters**	$\lambda_{1}$	$\lambda_{2}$	$G_{s}^{PBSWW}$	$G_{u}^{PBWSS}$
	3.32	2	0.85	1.12

**Table 5:** The used parameters for the PBWSS with the IT2FSC for dynamic unloading force control.

**Membership function of** $F^{i}$	$M\left(F^{i}\right)=\left[\left(c_{1},c_{2},\sigma \right)\right]=\left[\begin{array}{ccc} \left(-22,-20,1.5\right) & \left(-13,-11,1.5\right) & \left(-8.7,-6.7,1.5\right)\\ \left(-4.6,-2.6,1.5\right) & \left(-1,1,1.5\right) & \left(2.1,4.5,1.5\right)\\ \left(6.4,8.7,1.5\right) & \left(13,15,1.5\right) & \left(20,22,1.5\right) \end{array}\right]$			
**Membership function of** $y^{i}$	$M\left(y^{i}\right)=\left[\left(y_{l},y_{r}\right)\right]=\left[\begin{array}{ccc} \left(10,9.5\right) & \left(9.2,9\right) & \left(8.5,8\right)\\ \left(7.5,7\right) & \left(6.6,6\right) & \left(5.5,5\right)\\ \left(3.5,3\right) & \left(2.5,2\right) & \left(1.5,0\right) \end{array}\right]$			
**Control parameters**	$\lambda_{1}$	$\lambda_{2}$	$G_{s}^{PBSWW}$	$G_{u}^{PBWSS}$
	3.32	2	0.75	1.06

## References

[1] Chen G., Zhou Z., Vanderborght B., Wang N., Wang Q. (2016). Proxy-based sliding mode control of a robotic ankle-foot system for post-stroke rehabilitation. Adv. Robot..

[2] Andrea G., Martin S., Elisa P., Jan E., Michael E., Marko W., Ingeborg K.M., Giovanni C. (2007). Brain representation of active and passive hand movements in children. Pediatr. Res..

[3] Lukas J., Laura M.C., Peter W., Robert R., Lars M., Spyros K. (2014). Brain activation associated with active and passive lower limb stepping. Front. Hum. Neurosci..

[4] Zhavoronkova L.A., Boldyreva G.N., Kuptsova S.V., Sharova E.V., Smirnov A.S., Pronin I.N. (2017). fMRI responses of the brain during active and passive movements in left-handed subjects. Hum. Physiol..

[5] Finch L., Barbeau H., Arsenault B. (1991). Influence of Body Weight Support on Normal Human Gait: Development of a Gait Retraining Strategy. Phys. Ther..

[6] He J.P., Kram R., McMahon T.A. (1991). Mechanics of running under simulated low gravity. J. Appl. Physiol..

[7] Kram R., Domingo A., Ferris D.P. (1997). Effect of reduced gravity on the preferred walk-run transition speed. J. Exp. Biol..

[8] Dietz V., Colombo G., Jensen L. (1994). Locomotor activity in spinal man. Lancet.

[9] Hesse S., Malezic M., Schaffrin A., Mauritz K.H. (1995). Restoration of gait by combined treadmill training and multichannel electrical stimulation in nonambulatory hemiparetic patients. J. Rehabil. Med..

[10] Visintin M., Barbeau H., Bitensky N.K., Mayo N.E. (1998). A new approach to retrain gait in stroke patients through body weight support and treadmill stimulation. Stroke.

[11] Hesse S., Konrad M., Uhlenbrock D. (1999). Treadmill walking with partial body weight support versus floor walking in hemiparetic subjects. Arch. Phys. Med. Rehabil..

[12] Wernig A., Nanassy A., Mller S. (1999). Laufband (treadmill) therapy in incomplete paraplegia and tetraplegia. J. Neurotrauma.

[13] Edgerton V.R., Leon R.D., Harkema S.J., Hodgson J.A., London N., Reinkensmeyer D.J., Roy R.R., Talmadge R.J., Tillakaratne N.J., Timoszyk W. (2001). Retraining the injured spinal cord. J. Physiol..

[14] Hubertus J.A.H., Irene R., Sandra B.R. (2021). Clinical utility of the over-ground bodyweight-supporting walking system Andago in children and youths with gait impairments. J. Neuroeng. Rehabil..

[15] Grzegorz G., Piotr G., Slawomir D. (2021). Control System Design of an Underactuated Dynamic Body Weight Support System Using Its Stability. Sensors.

[16] Grzegorz G., Piotr G., Slawomir D. (2021). Modeling and Control of an Underactuated System for Dynamic Body Weight Support. Appl. Sci..

[17] Barbeau H., Visintin M. (2003). Optimal outcomes obtained with body-weight support combined with treadmill training in stroke subjects. Arch Phys. Med. Rehabil..

[18] Mehrholz J., Pohl M., Elsner B. (2014). Treadmill training and body weight support for walking after stroke. Cochrane Database Syst. Rev..

[19] Mao Y.R., Lo W.L., Lin Q., Li L., Xiao X., Raghavan P., Huang D.F. (2015). The effect of body weight support treadmill training on gait recovery, proximal lower limb motor pattern, and balance in patients with subacute stroke. BioMed Res. Int..

[20] Gazzani F., Fadda A., Torre M., Macellari V. (2000). WARD: A pneumatic system for body weight relief in gait rehabilitation. IEEE Trans. Rehabilitation Eng..

[21] Mcdaid A., Kora K., Xie S., Lutz J., Battley M. Human-inspired robotic exoskeleton (HuREx) for lower limb rehabilitation. Proceedings of the IEEE International Conference on Mechatronics and Automation.

[22] Riener R., Lunenburger L., Colombo G. (2006). Human-centered robotics applied to gait training and assessment. J. Rehabil. Res. Dev..

[23] Veneman J.F., Kruidhof R., Ekkelenkamp R. (2007). Design and evaluation of the LOPES exoskeleton robot for interactive gait rehabilitation. IEEE Trans. Neural Syst. Rehabilitation Eng..

[24] Fisher S., Lucas L., Thrasher T.A. (2011). Robot-assisted gait training for patients with hemiparesis due to stroke. Top. Stroke Rehabil..

[25] Guo B., Han J., Li X., Lin S.Y. (2019). Human–robot interactive control based on reinforcement learning for gait rehabilitation training robot. Int. J. Adv. Robot. Syst..

[26] Beyl P., Knaepen K., Duerinck S., Damme M.V., Vanderborght B., Meeusen R., Lefeber D. (2011). Safe and compliant guidance by a powered knee exoskeleton for robot-assisted rehabilitation of gait. Adv. Robot..

[27] Veale A.J., Xie S.Q. (2016). Towards Compliant and Wearable Robotic Orthoses: A Review of Current and Emerging Actuator Technologies. Med. Eng. Phys..

[28] Beyl P., Damme M.V., Ham R.V., Vanderborght B., Lefeber D. (2009). Design and control of a lower limb exoskeleton for robot-assisted gait training. Appl. Bionics Biomech..

[29] Ozkan M., Inou K., Negishi K., Yamanaka T. (2000). Defining a Neural Network Controller Structure for a Rubbertuator Robot. Neural Netw..

[30] Caldwell D.G., Tsagarakis N.G., Kousidou S., Costa N., Sarakoglou I. (2007). Soft exoskeletons for upper and lower body rehabilitation—Design, control and testing. Int. J. Hum. Comput. Stud..

[31] Lewek M.D. (2011). The influence of body weight support on ankle mechanics during treadmill walking. J. Biomech..

[32] Jiangtao C., Honghai L., Ping L., David B. Adaptive fuzzy logic controller for vehicle active suspensions with interval type-2 fuzzy membership functions. Proceedings of the IEEE International Conference on Fuzzy Systems.

[33] Bijan R.S., Mohammad S., Mehdi R. (2011). Control of Active Suspension System: An Interval Type -2 Fuzzy Approach. World Appl. Sci. J..

[34] Zirkohi M.M., Lin T.C. (2015). Interval type-2 fuzzy-neural network indirect adaptive sliding mode control for an active suspension system. Nonlinear Dyn..

[35] Chiu C.H., Hung Y.T. (2020). One wheel vehicle real world control based on interval type 2 fuzzy controller. Mechatronics.

[36] Kelekci E., Kizir S. (2019). Trajectory and vibration control of a flexible joint manipulator using interval type-2 fuzzy logic. ISA Trans..

[37] Barbeau H., Rossignol S. (1987). Recovery of locomotion after chronic spinalization in the adult cat. Brain Res..

[38] Hishikawa N., Tanikawa H., Ohtsuka K., Mukaino M., Inagaki K., Matsuda F., Teranishi T., Kanada Y., Kagaya H., Saitoh E. (2018). Quantitative assessment of knee extensor thrust, flexed-knee gait, insufficient knee flexion during the swing phase, and medial whip in hemiplegia using three-dimensional treadmill gait analysis. Top. Stroke Rehabil..

[39] Mukaino M., Ohtsuka K., Tsuchiyama K., Matsuda F., Inagaki K., Yamada J., Tanikawa H., Saitoh E. (2016). Feasibility of a Simplified, Clinically Oriented, Three-dimensional Gait Analysis System for the Gait Evaluation of Stroke Patients. Prog. Rehabil. Med..

[40] Li I.H., Lee L.W. (2012). Interval type 2 hierarchical FNN with the H-infinity condition for MIMO non-affine systems. Appl. Soft Comput..

[41] Tang Z., Xu X., Xiong J., Pei Z. (2015). Trajectory planning and mechanic’s analysis of lower limb rehabilitation robot. Biomed. Mater. Eng..

[42] Chen K., Liu Q., Wang R. (2011). Development of a body weight-support gait training robot. Chinese J. Rehabilitation Med..

